# Enzyme-Assisted Bacteria–Yeast Co-Fermentation of *Ganoderma lucidum*: Metabolomic Reprogramming, Sensory Evolution, and Sleep-Promoting Effects

**DOI:** 10.3390/foods15203716

**Published:** 2026-10-19

**Authors:** Yifan Wang, Yuxue Cui, Rongting Yang, Haoran Wu, An Zhou, Zimeng Ma, Yiping Song, Hui An, Yichang Chen, Huanhuan Ma, Rongxin Zou, Juan Liang

**Affiliations:** 1 School of Pharmacy, Anhui University of Chinese Medicine, Hefei 230012, China; 13953331569@163.com (Y.W.); 17730354201@163.com (Y.C.); yangrongt@outlook.com (R.Y.); 15163783968@163.com (H.W.); mzm1454821649@163.com (Z.M.); syp12333@outlook.com (Y.S.); 13412255236@163.com (H.A.); 13618536159@163.com (Y.C.); huanhuanma0923@163.com (H.M.); 2 Key Laboratory of Xin’an Medicine, Ministry of Education, Anhui University of Chinese Medicine, Hefei 230038, China; anzhou@ahtcm.edu.cn; 3 Functional Activity and Resource Utilization of Edible and Medicinal Fungi Anhui Provincial Joint Key Laboratory, Anhui University of Chinese Medicine, Hefei 230012, China; 4 Anhui Province Key Laboratory of Pharmaceutical Preparation Technology and Application, Hefei 230012, China

**Keywords:** *Ganoderma lucidum*, co-fermentation, untargeted metabolomics, sensory profile, gut microbiota, functional food

## Abstract

To enhance the extraction efficiency and bioactivity of *Ganoderma lucidum*, this study developed a novel enzyme-assisted bacteria–yeast co-fermentation system utilizing *Lactobacillus acidophilus* and *Saccharomyces cerevisiae*. Following optimization using response surface methodology, the maximum yields of polysaccharides and triterpenoids reached 2.07% and 3.55%, respectively, substantially exceeding those obtained by direct extraction. Structural characterization revealed that the integrated process induced substantial changes in the polysaccharides, including increased homogeneity, mannose enrichment, and a more porous microstructure. Furthermore, untargeted metabolomics demonstrated that co-fermentation significantly altered the small-molecule profile, characterized by the differential regulation of specific triterpenoids and the enrichment of peptides and amino acids. These metabolic shifts were associated with the evolution of the sensory profile, particularly increased taste richness. In vivo evaluations using a mouse model of PCPA-induced insomnia revealed that the fermented extract exhibited greater sleep-promoting activity than the direct extract. The fermented extract increased hypothalamic 5-HT and GABA levels and was accompanied by modulated gut microbiota composition by increasing the abundance of *Lactobacillus* and reducing that of *Prevotella*. These findings suggest that the combined enzymatic-fermentation strategy alleviates insomnia, potentially involving modulation of the microbiota–gut–brain axis, offering preliminary insights into the potential application of *Ganoderma lucidum* in functional foods.

## 1. Introduction

*Ganoderma* is the dried fruiting body of *Ganoderma lucidum* or *Ganoderma sinense*, two fungi belonging to the Polyporaceae family [1]. The 2025 edition of the *Chinese Pharmacopoeia* also states that *Ganoderma lucidum* “tonifies qi and calms the mind”. Research has indicated that extracts derived from *Ganoderma lucidum* exhibit diverse biological properties, notably immune system regulation, sleep enhancement, and neuroprotection. [2]. Their primary bioactive constituents include polysaccharides and triterpenes [3,4]. However, the dense cell wall structure of *Ganoderma lucidum* fruiting bodies can impede the release of bioactive constituents during conventional extraction, thereby reducing their extraction efficiency and bioavailability and limiting their potential pharmacological effects. In addition, compounds such as triterpenes in *Ganoderma lucidum* exhibit pronounced bitterness and astringency, which restrict their application in oral formulations and functional foods [5]. Therefore, developing effective biotransformation strategies for *Ganoderma lucidum* fruiting bodies is of considerable scientific and practical significance for facilitating the release and utilization of bioactive constituents and expanding their potential applications.

Microbial fermentation, a mild and efficient biotransformation technology, offers a promising approach to addressing these issues [6]. Probiotics can secrete enzymes, such as cellulases and glycosidases, that degrade cellular walls and facilitate the liberation of intracellular bioactive constituents [7]. Meanwhile, microorganisms can modify the molecular structures of bioactive constituents through biotransformation, thereby enhancing their bioactivity or generating novel functional compounds [8]. In addition, microbial metabolism can degrade precursors of bitter-tasting compounds and enhance the sensory quality of fermented products [9]. Strain selection is a key factor determining the efficiency and outcome of biotransformation. *Lactobacillus acidophilus* exhibits acid and bile salt tolerance and possesses acid-producing and bacteriostatic properties that contribute to improved fermentation-system stability [10]. Its extracellular enzymes can promote the degradation of proteins and polysaccharides, thereby increasing the contents of bioactive constituents, free amino acids, and small peptides [11]. *Saccharomyces cerevisiae* is characterized by its fast proliferation and robust stability. It can secrete various hydrolytic enzymes, promote the degradation of macromolecular substances, and improve nutrient utilization [12]. Furthermore, *S. cerevisiae* is rich in diverse bioactive components, which can enhance the nutritional value and antioxidant capacity of fermented products [13,14]. Co-fermentation with *L. acidophilus* and *S. cerevisiae* can exert synergistic effects by promoting the degradation of proteins and polysaccharides; increasing the release of bioactive constituents and improving sensory quality; facilitating the accumulation of flavor compounds; improving sensory quality; and enhancing antioxidant activity, functional constituent content, and fermentation-system stability [15].

In recent years, probiotic fermentation has gained increasing attention as a strategy for biotransforming traditional Chinese medicinal resources. Bai et al. fermented ginseng using *L. acidophilus* and found that the strain catalyzed the stepwise conversion of major ginsenosides, including Rb_1_, Rc, and Rd, into rare ginsenosides such as compound K, Rg_3_, and Rh_2_ through β-glucosidase activity, accompanied by enhanced antioxidant, anti-inflammatory, and antitumor activities [8]. Zhang et al. used *L. acidophilus* to ferment Astragalus membranaceus, which promoted the degradation of *Astragalus polysaccharides* and the production of bioactive oligosaccharides, thereby enhancing its immunomodulatory effects [7]. Similarly, Kim et al. fermented cinnamon with *S. cerevisiae* and reported higher levels of antioxidant constituents in the fermented product than in unfermented cinnamon, accompanied by an increase in anti-inflammatory activity from 89% to 92% [16]. In mixed-culture fermentation, Li et al. fermented an aqueous extract of *Ganoderma lucidum* fruiting bodies with *L. acidophilus* and Bifidobacterium breve [17]. Fermentation promoted the biotransformation of ganoderic acid A into ganoderic acid C2 and ameliorated dexamethasone-induced immunosuppression and gut microbiota dysbiosis in mice. Microbial fermentation offers a potential approach for improving the bioavailability of *Ganoderma lucidum* [18]. However, the dense cell wall structure composed of chitin and glucans often limits the efficiency of microbial enzymatic hydrolysis alone [19]. To overcome this barrier, an enzyme-assisted approach has been increasingly adopted. Pre-treatment with exogenous enzymes, such as cellulases and pectinases, can effectively degrade the fungal cell walls and release intracellular polysaccharides and triterpenoids into the medium. This provides readily accessible substrates for subsequent microbial metabolism, thereby facilitating deeper biotransformation and improving overall extraction yields. Therefore, combining exogenous enzymatic hydrolysis with bacteria–yeast co-fermentation represents a synergistic strategy [20]. Despite these advances, existing studies have primarily focused on the biotransformation and associated bioactivity changes in individual chemical constituents in traditional Chinese medicinal resources. Consequently, the overall transformation patterns of multiple classes of chemical constituents, changes in flavor quality, and relationships among these changes and bioactivities during fermentation remain insufficiently characterized. In addition, few studies have systematically integrated chemical composition, sensory quality, and functional activity into a multidimensional evaluation framework [21]. This gap limits a comprehensive understanding of the effects of microbial fermentation on the overall quality and biological functions of traditional Chinese medicinal resources.

To address these research gaps, this study used *Ganoderma lucidum* fruiting bodies as the fermentation substrate and established a dual-strain co-fermentation system comprising *L. acidophilus* and *S. cerevisiae*. The fermentation process was optimized, followed by systematic analysis of bioactive constituents, multidimensional assessment of product quality, and validation of sleep-promoting efficacy. The present work sought to advance the high-value utilization of *Ganoderma lucidum* fruiting bodies, provide a methodological framework for investigating the “composition–quality–efficacy” relationship in fermented products, and generate a process foundation and supporting data for developing *Ganoderma lucidum*-based health products with sleep-promoting properties.

## 2. Materials and Methods

### 2.1. Materials and Reagents

*Ganoderma lucidum* fruiting bodies were provided by Anhui Jinzhai Qiaokang Pharmaceutical Co., Ltd. (Lu’an, China). The strains used in this study were *Lactobacillus acidophilus* (CGMCC 1.1854) and *Saccharomyces cerevisiae* (CGMCC 2.3868), both sourced from the China General Microbiological Culture Collection Center in Beijing, China. Bovine serum albumin (BSA), Coomassie brilliant blue, and Tween-80 were purchased from Biosharp Biotechnology Co., Ltd. (Hefei, China). Monosaccharide standards (e.g., rhamnose and glucose) were sourced from Sigma-Aldrich (St. Louis, MO, USA). Chromatographic-grade reagents, including formic acid, trifluoroacetic acid (TFA), D_2_O, and LC-MS grade methanol, were obtained from Thermo Fisher Scientific (Waltham, MA, USA), ANPEL (Shanghai, China), and Macklin Biochemical Co., Ltd. (Shanghai, China). YPD and MRS culture media were purchased from Haibo Biotechnology Co., Ltd. (Qingdao, China). All remaining chemicals and enzymatic preparations met analytical-grade purity requirements.

### 2.2. Optimization of Ganoderma lucidum Fermentation

#### 2.2.1. Strain Activation and Inoculum Preparation

Lyophilized strains were resuscitated and cultured in LB (30 °C), MRS (37 °C), and YPD (25 °C) liquid media, respectively. After purification on solid media, single colonies were selected to prepare bacterial/fungal suspensions. The optical density (OD_600_) of each suspension was set to 0.4. A dual-strain co-fermentation inoculum was formed by mixing *L. acidophilus* and *S. cerevisiae* at a 1:1 (*v*/*v*) ratio.

#### 2.2.2. Single-Factor Experiments

The impacts of fermentation duration, temperature, starting pH, and inoculum level on the yields of total *Ganoderma lucidum* polysaccharides (GLPs) and triterpenes (GLTs) were investigated through individual-factor experiments. Each experiment involved changing a single factor while maintaining the remaining variables at fixed levels (Table S1).

#### 2.2.3. Response Surface Methodology (RSM) Optimization

Guided by the findings of the single-factor experiments., a Box–Behnken Design (BBD) was utilized to refine the fermentation conditions. Three independent variables-fermentation time (A), initial pH (B), and inoculum size (C)-were assessed at three levels (Table S2). Regression analysis was performed to determine and validate the optimal co-fermentation conditions.

### 2.3. Extraction of Polysaccharides and Triterpenes

#### 2.3.1. Direct Extraction (DE)

Following a modified method [22], 10.0 g of *Ganoderma lucidum* powder underwent extraction using hot water (90 °C), using a 1:20 solid-to-liquid proportion over a 6 h extraction period. The supernatant was collected via centrifugation, precipitated by adding absolute ethanol at a fourfold volume, centrifuged again, followed by freeze-drying to yield the crude direct-extracted polysaccharide (GLP-DE). The remaining residue was extracted with 90% ethanol at 70 °C for 2 h. The filtrate was subjected to vacuum concentration followed by freeze-drying to yield the direct-extracted triterpenes (GLT-DE). Quantification of GLP and GLT was carried out using the phenol–sulfuric acid assay and the oleanolic acid–perchloric acid assay, respectively. The calculation formulas are expressed as follows:

(1) $$\text{Yiel}d_{\text{GLP}}\left(\%\right)=\frac{{\text{Mass}}_{\text{GLP}}}{{\text{Mass}}_{\text{Raw Powder}}}\times 100\%$$

(2) $$\text{Yiel}d_{\text{GLT}}\left(\%\right)=\frac{{\text{Mass}}_{\text{GLT}}}{{\text{Mass}}_{\text{Raw Powder}}}\times 100\%$$

Mass_GLP_ refers to the polysaccharide content calculated using the standard curve, Mass_GLT_ refers to the triterpenoid content calculated using the standard curve, and Mass_Raw Powder_ refers to the mass of *Ganoderma lucidum* fruiting body powder.

#### 2.3.2. Probiotic Fermentation Extraction (PF)

*Ganoderma lucidum* powder (10.0 g) was mixed with acetate buffer (pH 5.5) at a 1:20 ratio. Enzymatic hydrolysis was carried out with cellulase at 50 °C over a 2 h period, pectinase, and papain, each at a dosage of 2% (*w*/*v*). The enzymes were subsequently inactivated through thermal treatment at 90 °C for 10 min. After sterilization (121 °C, 15 min), the mixture was inoculated with 2.3% (*v*/*v*) of the *L. acidophilus-S. cerevisiae* mixed suspension and fermented at 30 °C with an initial pH of 5.1 for 27 h. Post-fermentation, GLP-PF and GLT-PF were extracted following the same centrifugation, ethanol precipitation, and lyophilization procedures described in Section. GLP and GLT yields were calculated according to Equations (1) and (2), as described in Section 2.3.1.

### 2.4. Physicochemical Properties and Preliminary Structural Analysis of Crude Polysaccharide Fractions

#### 2.4.1. Chemical Composition Analysis

Crude polysaccharides (GLP-DE and GLP-PF) were deproteinized using the Sevag method [23]. Total sugar content was quantified colorimetrically according to the phenol–sulfuric acid procedure, with glucose used to generate the calibration curve. Uronic acid content was determined through the m-hydroxydiphenyl colorimetric reaction. Protein levels were measured using the Bradford protein assay, with bovine serum albumin (BSA) as the calibration reference. The overall triterpenoid concentration was quantified using a colorimetric assay based on the vanillin–perchloric acid reaction [24]. It should be noted that the Sevag deproteinization method effectively removes free proteins but may not completely eliminate bound proteins or microbial cellular debris. Therefore, GLP-DE and GLP-PF are considered crude polysaccharide complexes containing residual non-carbohydrate materials. The total carbohydrate content was calculated according to the following equation:

(3) $$\text{Total}\;\text{sugar}\;\text{content}\left(\%\right)=\frac{\text{Mas}s_{\text{GLP}}}{\text{Mas}s_{\text{lyophilized}\;\text{extract}}}\times 100\%$$

Mass_GLP_ refers to the polysaccharide content calculated using the standard curve, and Mass_lyophilized_ extract refers to the mass of the crude extract obtained after ethanol precipitation and freeze-drying.

#### 2.4.2. Molecular Weight Distribution

An Agilent 1260 liquid chromatograph coupled with a DEAC901030 refractive index detector (Agilent Technologies, Santa Clara, CA, USA) was utilized to characterize the molecular weight distribution of the samples. The samples were analyzed using a TSK gel G4000PWX1 column (Tosoh Corp., Tokyo, Japan) maintained at 35 °C. Elution was performed with ultrapure water at 0.6 mL/min, and 10 µL of each sample was injected for analysis [25]. A standard curve was constructed using dextran reference standards spanning molecular weights of 6, 40, 100, 500, and 2000 kDa.

#### 2.4.3. Monosaccharide Composition

Polysaccharide samples underwent acid hydrolysis with 2 M TFA at 121 °C over a 2 h period. Following nitrogen-stream evaporation, the residues were redissolved in sterile water. Monosaccharide composition was determined by ion chromatography on a Thermo Fisher Scientific ICS 5000+ system (Thermo Fisher Scientific, Waltham, MA, USA) with electrochemical detection and a Dionex CarboPac PA20 column (150 × 3.0 mm, 10 μm) (Thermo Fisher Scientific, Waltham, MA, USA). Elution employed H_2_O, 0.1 M NaOH, and 0.1 M NaOH containing 0.2 M NaAc at 0.5 mL/min and 30 °C [26].

#### 2.4.4. FT-IR Spectroscopy and SEM Morphology

Dried polysaccharides were blended with KBr at a 1:100 sample-to-KBr ratio and compressed into 2 mm pellets. Infrared spectral data were acquired on an FT-IR spectrometer using a spectral resolution of 4 cm^−1^ and averaging 32 scans per measurement collected for each measurement [27]. For morphological observation, samples were sputter-coated with gold and examined under a Zeiss Sigma 300 scanning electron microscope (SEM) (Carl Zeiss, Jena, Germany) at an accelerating voltage of 5 kV [28].

#### 2.4.5. Nuclear Magnetic Resonance (NMR) Analysis

Lyophilized GLP-DE and GLP-PF (30 mg each) were solubilized in 0.55 mL of D_2_O. Proton nuclear magnetic resonance (^1^H NMR), carbon-13 nuclear magnetic resonance (^13^C NMR), and heteronuclear single-quantum correlation (HSQC) spectra were acquired using a Bruker AVANCE NEO 600 MHz spectrometer (Bruker, Rheinstetten, Germany) at 60 °C [29].

### 2.5. Untargeted Metabolomic Analysis of Ethanol Extracts

The small-molecule compositions of the ethanol extracts (SM-DE and SM-PF) were analyzed using LC-MS/MS. Lyophilized powders (equivalent to extracts from 20 mg of raw powder) were dissolved in 100 μL of methanol, vortexed, and centrifuged (12,000 r/min, 5 min). The supernatant was filtered (0.22 μm) for analysis. A QC sample was prepared by pooling 10 μL from each sample. During sample injection, a QC sample was inserted into the sequence at intervals of every four samples; the relative standard deviation (RSD) values were calculated based on the peak areas and retention times of the characteristic ions to assess sample stability. The QC sample was injected six times consecutively under the experimental conditions described above to select characteristic ion peaks and simultaneously calculate the relative standard deviation (RSD) of their peak areas and retention times to assess the instrument’s precision.

An Agilent Eclipse Plus C18 column (Santa Clara, CA, USA) was used for chromatographic separation. (2.1 × 100 mm, 1.8 μm). Chromatographic elution was carried out at 0.3 mL/min using aqueous formic acid (0.1%, solvent A) and methanol (solvent B). The gradient elution was: 0–3 min, 60–45% A; 3–10 min, 45–35% A; 10–28 min, 35–5% A; 28–38 min, 5% A; and 38.1–42 min, 60% A.

High-resolution MS was conducted in both ESI(+) and ESI(−) modes. Parameters included: a mass range of 120–1000 m/z, spray voltage of 3.2 kV (+)/2.8 kV (−), and capillary temperature of 325 °C. Data acquisition utilized Full MS/dd-MS^2^ mode at a resolving power setting of 60,000. Supervised orthogonal partial least squares discriminant analysis (OPLS-DA) combined with univariate statistical analysis (Student’s *t*-test) was used to screen differential metabolites among the different groups. Differential features were selected using the criteria of *p* < 0.05 and VIP scores exceeding 1. For initial compound annotation, measured precursor-ion m/z values were queried against the Human Metabolome Database (HMDB; [http://www.hmdb.ca/]), accepting mass deviations within ±10.0 ppm. Subsequently, the MS_1_ and MS_2_ fragmentation information acquired by UPLC-QE/MS was compared with metabolite information in online databases for further verification of the differential metabolites. Then, the identified differential metabolite data were imported into MetaboAnalyst 5.0 in CSV format, and taste-profile correlation analysis was performed to screen metabolites significantly associated with taste characteristics, thereby identifying the key differential metabolites. Finally, Compound Discoverer 3.3 was used to perform feature peak extraction, peak alignment, and normalization of the raw UPLC-QE/MS data. Candidate compounds were matched against databases such as HMDB, mzCloud, and ChemSpider based on accurate mass and MS/MS information, and further compared and identified by integrating relevant literature and characteristic MS/MS fragmentation patterns [23].

### 2.6. Electronic Tongue (Taste) Analysis

Sensory profiles were evaluated using an electronic tongue (SA402BPlus, Insent Inc., Atsugi, Kanagawa, Japan). Sensors (C1–C5) were activated in a tartaric acid-KCl reference solution for 30 min. Each test cycle included a 120 s wash phase and a 180 s measurement phase. Each sample was tested four times. Data from the last three stable cycles were analyzed for significance using SPSS, and radar charts were plotted using Origin 2021 software [30].

### 2.7. Evaluation of Sleep-Promoting Efficacy

#### 2.7.1. Animal Experimental Design

Fifty-six SPF male ICR mice (18–22 g) were sourced from Hangzhou Ziyuan Experimental Animal Technology Co., Ltd. (Hangzhou, China) (License: SCXK(Zhe)2024-0004). The mice were maintained under specific pathogen-free (SPF) housing conditions, with the temperature controlled at a temperature of 22 ± 2 °C and a humidity level of maintained at 50 ± 5%. All procedures were reviewed and authorized by the Animal Ethics Committee of Anhui University of Chinese Medicine (Approval No. AHUCM-mouse-2026043). Following one week of acclimation, the animals were allocated at random to seven experimental groups (n = 8) using a computer-generated randomization sequence: Normal Group (NG), Model Group (PCPA), Positive Control Group (DZP), and four treatment groups (DE-L, DE-H, PF-L, PF-H). To establish the insomnia model, mice in all groups except the normal control (NG) received daily intraperitoneal injections of p-chlorophenylalanine (PCPA) at 500 mg/kg for three successive days. Subsequently, the DZP group received diazepam suspension (2.5 mg/kg) via oral gavage, while treatment groups received corresponding extracts (1 mL/kg). NG and PCPA groups received equal volumes of saline. All treatment regimens were continued once daily over a 6-day period. The experiment was conducted under blinded conditions.

The animals received *Ganoderma lucidum* preparations containing polysaccharides, triterpenoids, and other extractable constituents. Briefly, 400.0 g of *G. lucidum* fruiting-body powder was used to prepare the high-dose aqueous extract (DE-H), which was concentrated to 40 mL, while DE-L was prepared by two-fold dilution of DE-H. Similarly, 400.0 g of *G. lucidum* fruiting-body powder was fermented under the optimized conditions (30 °C, 27 h, initial pH 5.1, and probiotic inoculum 2.3%), and the resulting supernatant was concentrated to 40 mL to obtain PF-H. PF-L was prepared by two-fold dilution of PF-H. The dose levels were determined based on our research group’s previous experimental experience**.**

#### 2.7.2. Sleep Behavior and Sample Collection

Thirty minutes after the final gavage, all mice were intraperitoneally injected with pentobarbital sodium (65 mg/kg) to record sleep latency and duration [7,31]. Following a 12 h fast, mice were anesthetized. Hypothalamic tissues were rapidly isolated on ice; a portion underwent fixation in 4% paraformaldehyde for histopathology, and the remainder was maintained at −80 °C for neurotransmitter assays. Colon contents were collected for microbiome analysis.

#### 2.7.3. Biochemical and Histopathological Analysis

Levels of 5-HT, GABA, and Glu in hypothalamic tissues were quantified using commercial ELISA kits according to the manufacturer’s instructions. For histological examination, the fixed hypothalamic samples underwent sequential dehydration in graded ethanol, followed by paraffin infiltration and preparation of 5 μm-thick sections. The resulting sections were subsequently subjected to hematoxylin and eosin (H&E) staining. Pathological morphological changes were observed under a light microscope.

#### 2.7.4. Gut Microbiota Analysis (16S rRNA Sequencing)

Genomic DNA was isolated from colon contents using the OMEGA Soil DNA Kit (D5635-02, Omega Bio-Tek, Norcross, GA, USA). DNA integrity and purity were evaluated by agarose gel electrophoresis and NanoDrop spectrophotometry. The V3–V4 hypervariable segment of the bacterial 16S rRNA gene was targeted using the 338F primer pair (5′-ACTCCTACGGGAGGCAGCAG-3′)/806R (5′-GGACTACNNGGGTATCTAAT-3′). Sequencing libraries were then analyzed on an Illumina NovaSeq 6000 instrument (Illumina, San Diego, CA, USA), generating 250-base reads from both ends of each fragment (2 × 250 bp). Raw sequencing data were subsequently processed with Cutadapt and DADA2 to remove primers, filter low-quality reads, perform denoising, merge paired-end sequences, and eliminate chimeric sequences. ASVs with a total abundance of one were removed. QIIME 2 was used to assign taxonomic identities to the sequences, applying a minimum classifier confidence score of 0.8 and referencing either Greengenes (version 13.8) or SILVA (version 138) for taxonomic annotation. The DADA2 plugin in QIIME 2 was used for quality control and ASV generation. Alpha and beta diversity analyses were performed using the Oebiotech platform.

### 2.8. Statistical Analysis

Statistical analyses were executed using IBM SPSS Statistics (version 27), and results are represented by mean ± standard deviation ($\bar{x}\pm s$). Comparisons between two independent groups were assessed by Student’s unpaired *t*-test, whereas differences across multiple groups were evaluated by one-way ANOVA. Longitudinal body weight measurements were examined using two-way repeated-measures ANOVA. Different lowercase letters denote significant differences among groups (*p* < 0.05), while identical letters indicate the absence of statistically significant differences. A *p*-value below 0.05 was considered indicative of a statistically significant difference.

## 3. Results

### 3.1. Optimization of the Fermentation Process of Ganoderma lucidum Fruiting Bodies via Response Surface Methodology

Single-factor experiments were initially conducted to evaluate the effects of fermentation time, temperature, initial pH, and inoculum size on the yields of total polysaccharides and total triterpenoids from *Ganoderma lucidum* fruiting bodies (Figure 1). Fermentation time, initial pH, and inoculum size significantly influenced the extraction yields, whereas temperature exhibited a marginal effect. Specifically, the yields of both target components initially increased and subsequently decreased with increasing fermentation time and initial pH. Furthermore, increasing the inoculum size promoted triterpenoid accumulation but decreased polysaccharide yield. Based on these findings, fermentation time (a), initial pH (b), inoculum size (c), and fermentation temperature (d) were selected as independent variables for single-factor experiment, with fermentation temperature fixed at 30 °C.

The fermentation process was further optimized using a Box–Behnken experimental design. The experimental design matrix and the corresponding yields of total polysaccharides and total triterpenoids are presented in Table S3. Total polysaccharide yield (Y_1_) and total triterpenoid yield (Y_2_) were used as the response variables, and the experimental data were fitted to quadratic regression models. The analysis of variance results for the regression models of total polysaccharide and total triterpenoid yields are shown in Table 1 and Table 2, respectively. Both response surface models were statistically significant (*p <* 0.05), whereas the lack-of-fit terms were not significant (*p >* 0.05), indicating that the models adequately fitted the experimental data and were suitable for describing the relationships between the independent variables and the corresponding responses.

For the total polysaccharide yield model, the relative effects of the factors on the response followed the order of inoculum size (C) > initial pH (B) > fermentation time (A). The interaction between initial pH and inoculum size significantly influenced total polysaccharide yield. For the total triterpenoid yield model, the relative effects of the factors followed the order of initial pH (B) > fermentation time (A) > inoculum size (C). Quadratic multiple regression analysis yielded equations describing the relationships between the investigated factors and total polysaccharide and total triterpenoid yields:

Y_1_ = 0.124139A + 3.3475B − 0.236625C − 0.009444AB − 0.001111AC + 0.065BC − 0.001417A^2^ − 0.319B^2^ − 0.016813C^2^ − 7.75831 (4)

Y_2_ = 0.522583A + 9.6175B + 0.337375C − 0.000556AB + 0.0025AC − 0.04BC − 0.009639A^2^ − 0.923B^2^ − 0.035813C^2^ − 28.65881 (5)

For total polysaccharides, the quadratic model was significant (*F* = 11.27, *p* = 0.0021), with a non-significant lack of fit (*F* = 2.37, *p* = 0.2120), R^2^ = 0.9355, adjusted R^2^ = 0.8525, and predicted R^2^ = 0.3031. For total triterpenoids, the quadratic model was highly significant (*F* = 71.31, *p* < 0.0001), with a non-significant lack of fit (*F* = 5.07, *p* = 0.0755), R^2^ = 0.9892, adjusted R^2^ = 0.9753, and predicted R^2^ = 0.8598. These results provide additional information regarding the adequacy and predictive performance of the fitted models. Model predictions indicated that, when total polysaccharide yield was designated as the optimization objective, the optimal fermentation conditions were a fermentation time of 26.271 h, an initial pH of 5.047, and an inoculum size of 1.850%, with a predicted total polysaccharide yield of 2.100%. When total triterpenoid yield was designated as the optimization objective, the optimal conditions were a fermentation time of 27.323 h, an initial pH of 5.141, and an inoculum size of 2.793%, with a predicted total triterpenoid yield of 3.674%. Considering the yields of both total polysaccharides and total triterpenoids, together with practical production feasibility, the optimal fermentation conditions were determined to be a fermentation time of 27 h, an initial pH of 5.1, and an inoculum size of 2.3%. Under these conditions, the predicted yields of total polysaccharides and total triterpenoids were 2.100% and 3.674%, respectively.

The optimized process was experimentally validated, yielding actual total polysaccharide and total triterpenoid values of 2.07 ± 0.08% and 3.55 ± 0.18%, with prediction errors of only 1.24% and 3.09%, respectively. The absence of significant differences between the predicted and observed values (*p* > 0.05) confirmed the high reliability and predictability of the response surface models for *Ganoderma lucidum* fermentation. Furthermore, compared with the conventional extraction (DE group: 0.44 ± 0.02% for polysaccharides and 0.69 ± 0.05% for triterpenoids), the optimized probiotic fermentation (PF group) led to a 4.7-fold and 5.1-fold increase in these bioactive components, respectively *(p* < 0.001), highlighting the superior extraction efficacy of the co-fermentation process.

### 3.2. Impact of Fermentation on Physicochemical Properties of Crude Polysaccharide Complexes

#### 3.2.1. Chemical Composition Analysis

The standard curves for total sugar, uronic acid, and protein all exhibited excellent linearity (R^2^ > 0.99). The chemical compositions of GLP-DE and GLP-PF are presented in Table 3. The total sugar content of GLP-PF (34.67 ± 0.73%) was lower than that of GLP-DE (48.90 ± 3.96%). In contrast, co-fermentation increased the uronic acid content from 5.63% to 8.93% and the protein content from 0.90% to 1.44%.

#### 3.2.2. Molecular Weight Distribution

High-performance gel permeation chromatography was applied to characterize the molecular mass profiles of GLP-DE and GLP-PF, as illustrated in Figure 2a,b. The resulting calibration equation was: y = −0.6737x + 8.1245 (R^2^ = 0.9922). According to this calibration equation, GLP-DE exhibited a heterogeneous Mw distribution with three distinct fractions at 24.03, 7.87, and 2.42 kDa. In contrast, GLP-PF showed a more uniform and narrower Mw distribution, with two closely related fractions at 5.51 kDa and 5.86 kDa. The significant decrease in Mw and narrowing of the distribution indicate that the high-molecular-weight polysaccharides were effectively depolymerized and degraded during fermentation, likely due to microbial enzymatic activity.

#### 3.2.3. Monosaccharide Composition

Ion chromatography (Figure 2c–e) revealed that both GLP-DE and GLP-PF contained nine monosaccharides, including fucose (Fuc), rhamnose (Rha), arabinose (Ara), glucosamine (GlcN), galactose (Gal), glucose (Glc), xylose (Xyl), mannose (Man), and glucuronic acid (Glc-UA). Relative proportions of these monosaccharides are presented in Table S4. Glc was the predominant monosaccharide in both samples, accounting for more than 50% of the total. Notably, fermentation altered the monosaccharide composition, with the relative contents of Fuc and Gal significantly decreasing in GLP-PF, whereas the relative content of Man significantly increased. These changes may reflect the selective utilization or biotransformation of specific polysaccharide components by the probiotic strains during fermentation.

#### 3.2.4. Fourier Transform Infrared (FT-IR) Spectroscopic Analysis

The FT-IR spectra (Figure 2f) demonstrated highly similar absorption profiles for GLP-DE and GLP-PF across the scanned range. The broad, intense band at 3345.89 cm^−1^ was attributed to O–H stretching vibrations associated with intermolecular and intramolecular hydrogen bonding. The absorption bands at 2942.84 and 1371.14 cm^−1^ were attributed to C–H stretching and bending vibrations, respectively. The peak at 1664.27 cm^−1^ was attributed to residual bound water and/or C=O stretching vibrations. Collectively, these characteristic absorption bands were consistent with the characteristic functional groups of polysaccharides. The bands at 1604.48 and 1430.92 cm^−1^ were attributed to asymmetric and symmetric COO^−^ stretching vibrations, respectively, indicating the presence of uronic acid residues in both samples. Additionally, the signals at 1159.01, 1081.87, 1025.94, and 565.04 cm^−1^ were associated with C–O–C glycosidic linkages and pyranose ring vibrations. The high spectral similarity between GLP-DE and GLP-PF indicated that fermentation did not substantially alter the primary functional groups of the polysaccharides.

#### 3.2.5. Micromorphological Analysis

The surface morphologies of the crude polysaccharide powders were examined using scanning electron microscopy (Figure 2g–j). GLP-DE exhibited a relatively compact surface with protrusions but few visible pores or cracks. In contrast, the GLP-PF powder displayed a looser three-dimensional network composed of numerous small fragments, characterized by distinct cracks and porous structures. These visual differences suggest that the combined enzymatic and fermentation process resulted in a more disrupted physical matrix compared to direct extraction.

#### 3.2.6. NMR Spectroscopic Analysis

Structures of GLP-DE and GLP-PF were characterized using ^1^H, ^13^C, and HSQC NMR spectroscopy (Figure 3). In the ^1^H NMR spectra, the proton signals of the polysaccharides were predominantly observed between δ 3.0 and 6.0 ppm. Anomeric proton signals characteristic of β-glycosidic linkages, typically observed at δ 4.3–4.8 ppm, were observed at δ 4.41 ppm in GLP-DE and δ 4.45 ppm in GLP-PF. Additional signals in the δ 4.90–5.18 ppm range, which are consistent with α-glycosidic linkages, were observed in both samples, suggesting the presence of both α- and β-configurations. Methyl proton signals characteristic of 6-deoxy sugars, typically observed at δ 1.1–1.3 ppm, appeared at δ 1.09 in GLP-DE and δ 1.20 in GLP-PF. The sharper, more intense Fuc-related signals in GLP-DE were consistent with the higher relative Fuc content observed in GLP-DE compared with GLP-PF. A weak signal at δ 5.23 ppm, assigned to the H-1 proton of Man, was observed in GLP-PF at δ 5.22 ppm but was not detected in GLP-DE, suggesting the presence of Man in GLP-PF. The residual solvent signal appeared at δ 4.71 ppm.

In the ^13^C NMR spectra, anomeric carbon signals were distributed between δ 95 and 110 ppm. Both polysaccharides showed a characteristic signal at δ 16.75 ppm, which was assigned to C-6 of α-L-Fuc residues [32]. Intense signals in the δ 59.95–61.00 ppm range were attributed to C-5 of Ara and Xyl and/or C-6 of Man [33]. Signals at δ 15.51 ppm in GLP-DE and δ 15.50 ppm in GLP-PF were consistent with methyl or 6-deoxy sugar residues. Notably, GLP-DE showed a distinct acetyl methyl signal at δ 22.08 ppm that was absent in GLP-PF, suggesting the presence of acetylated groups in GLP-DE [32].

The HSQC spectra revealed cross-peaks corresponding to Galp residues at δH/δC 4.98/97.88 ppm in GLP-DE and δH/δC 4.88/97.88 ppm in GLP-PF. Furthermore, both samples showed a cross-peak at δH/δC 4.68/102.72 ppm, consistent with a β-glycosidic linkage and supporting the ^1^H NMR findings [34].

The NMR spectra provided preliminary information on the chemical environments of the carbohydrate moieties and the characteristics of the anomeric carbons in GLP-DE and GLP-PF. The observed signals indicated differences in the carbohydrate chemical environments between the two samples, particularly regarding the presence of acetyl groups and Man residues.

### 3.3. Characterization of Small-Molecule Components in Ganoderma lucidum Fermentation Broth

Untargeted metabolomic profiling of SM-DE and SM-PF was performed using UPLC-QE/MS in both positive and negative ionization modes, with representative total ion chromatograms (TICs) shown in Figure S1. The analysis revealed distinct metabolic profiles between the two groups. Differential putative metabolites were initially screened using thresholds of |log_2_FC |> 1 and *p* < 0.05, with putative metabolite identifications based on the HMDB database and MS/MS fragmentation data. Volcano plot analysis (Figure S2) identified 3034 upregulated and 1916 downregulated putative metabolites in SM-PF compared with SM-DE. Principal component analysis (PCA; Figure 4) demonstrated tight clustering of quality control (QC) samples and clear separation between the two experimental groups. This distinction was further supported by orthogonal partial least-squares discriminant analysis (OPLS-DA; Figure 4), which yielded a robust model (R^2^Y and Q^2^ > 0.5). Applying the more stringent criteria of Variable Importance in Projection (VIP) ≥ 1 and *p* < 0.05, 19 key differential putative metabolites were identified between the two groups.

Metabolite classification (Table S5) identified 98 differential compounds between the directly extracted (SM-DE) and fermented (SM-PF) groups, primarily comprising terpenoids, peptides and amino acids, fatty acids, flavonoids, coumarins, and alkaloids. These metabolite classes exhibited distinct patterns of change following fermentation. Terpenoids were predominantly decreased, with three compounds, including lucidenic acid B, upregulated and five compounds, including ganoderic acid E, downregulated. In contrast, peptides and amino acids were predominantly enriched, with 12 putative metabolites, including L-norleucine and glycylleucine, upregulated and only N-butyryl-L-homoserine lactone downregulated. Fatty acid derivatives exhibited a similar pattern, with seven putative metabolites, including N-lauroylglycine and palmitoylcarnitine, upregulated and four compounds, including DL-carnitine and phenylacetylglycine, downregulated. Flavonoids and coumarins were predominantly decreased, with 14 flavonoids, including phloretin and naringenin, and eight coumarins, including byakangelicol and osthole, downregulated, compared with only one flavonoid, aspalathin, and two coumarins, byakangelicin and 8,8-dimethyl-2H,8H-pyranocoumarin, upregulated. Finally, alkaloids showed an overall increase in abundance, with four compounds, including harmine and emetine, upregulated and papaverine downregulated.

### 3.4. Taste Analysis of Ganoderma lucidum Fermentation Broth

Electronic tongue analysis was performed to characterize the taste profiles of different *Ganoderma lucidum* extracts, using the washing solution as the blank control and setting the baseline response to zero. Aftertaste-A and Aftertaste-B correspond to astringent and bitter aftertastes, respectively. Higher positive response values indicate greater intensity of the respective taste attribute, whereas negative values indicate its absence. PCA demonstrated clear separation between the DE and PF groups (Figure 5a), indicating distinct overall taste profiles. Radar plots and statistical analyses (Figure 5b,c) indicated that sourness, saltiness, and astringency remained weak and statistically comparable across groups. Compared with the directly extracted samples, PF increased overall taste complexity. Collectively, these findings demonstrate that probiotic fermentation substantially altered the sensory profile of *Ganoderma lucidum*.

Pearson correlation analysis was performed to investigate the relationships between small-molecule putative metabolites and sensory attributes (Figure 5d). Umami showed a significant positive correlation with terpenoids (AKBA and oleanolic acid), peptides and amino acids (L-tyrosylglycylglycyl-L-tryptophyl-L-leucine, 5-hydroxytryptophan, DL-β-leucine, and L-phenylalanine), fatty acid derivatives (methylsuccinic acid and myristoyl-Gly-OH), the flavonoid retusin, and the alkaloid cephaeline (*p* < 0.001). In contrast, umami intensity was significantly negatively correlated with terpenoids phytonadiol, lucidenic acid B, and (+)-absinthin (*p* < 0.001), whereas taste richness was significantly negatively correlated with ganoderic acid B (*p* < 0.001). Bitterness was significantly positively correlated with 8′-apocapsorbinal, apocarotenal, and (+)-absinthin (*p* < 0.001) but negatively correlated with AKBA, 5-hydroxytryptophan, DL-β-leucine, L-phenylalanine, myristoyl-Gly-OH, L-tyrosylglycylglycyl-L-tryptophyl-L-leucine, retusin, oleanolic acid, and cephaeline (*p <* 0.001). Similarly, bitter aftertaste (Aftertaste-B) showed a significant positive correlation with cembrene A, 8′-apocapsorbinal, and apocarotenal (*p* < 0.001), and a significant negative correlation with ganoderic acid B (*p* < 0.001).

### 3.5. Sleep-Promoting Efficacy of Co-Fermented Ganoderma lucidum Extract

#### 3.5.1. Effects on Body Weight and Sleep Behavior in Mice with PCPA-Induced Insomnia

To evaluate the sleep-promoting effects of the extracts, a mouse model with PCPA-induced insomnia was established. Longitudinal body weight changes were analyzed using a two-way repeated-measures ANOVA, which revealed significant main effects for Treatment (*F*_(6, 49)_ = 3.556, *p* < 0.01) and Time (*F*_(2.714, 133)_ = 58.2, *p* < 0.0001), along with a significant Time × Treatment interaction (*F*_(30, 245)_ = 1.687, *p* = 0.0173). As shown in Figure 6a, the groups did not differ significantly in initial body weight. Following PCPA administration, the model-related groups exhibited a significant reduction in body weight compared with the normal group (NG, *p* < 0.05). However, the significant interaction effect confirmed that the recovery trajectories diverged depending on the intervention (Figure 6b). After 6 days of treatment, body weights in the DZP, DE-H, and PF-H groups recovered significantly (*p* < 0.05), with no significant differences among these three groups, whereas the low-dose groups (DE-L and PF-L) showed only marginal changes. The growth trend (Figure 6b) indicated that high-dose *Ganoderma lucidum* extracts effectively ameliorated PCPA-induced body weight loss.

A pentobarbital-induced sleep test was conducted to assess sleep latency and sleep duration. Compared with the NG, the PCPA model group exhibited significantly prolonged sleep latency and shortened sleep duration (*p* < 0.05), supporting the successful establishment of the insomnia model. As shown in Figure 6c,d, treatment with both DE and PF, as well as the positive control (DZP), significantly reduced sleep latency and prolonged sleep duration compared with the PCPA group (*p* < 0.05). Although sleep latency did not differ significantly among the extract-treated groups, the PF-H group showed the longest sleep duration. Sleep duration in the PF-H group was significantly longer than that in the low-dose groups and the DZP group (*p* < 0.05), as well as that in the DE-H group (*p* < 0.05). These findings suggest that probiotic co-fermentation enhanced the sleep-promoting effects of the *Ganoderma lucidum* extract in mice with PCPA-induced insomnia.

#### 3.5.2. Modulation of Hypothalamic Neurotransmitters and Histopathology

Neurotransmitters play an important role in the regulation of the sleep–wake cycle. To investigate potential mechanisms underlying the observed sleep-promoting effects, the levels of 5-HT, GABA, and Glu, as well as the GABA/Glu ratio, were measured in the hypothalamus (Figure 6e–h). PCPA administration altered hypothalamic neurotransmitter levels, as evidenced by significant decreases in 5-HT and GABA levels and in the GABA/Glu ratio (*p* < 0.05), whereas Glu showed a nonsignificant increasing trend. Treatment with DZP, DE, and PF significantly increased 5-HT and GABA levels and the GABA/Glu ratio (*p* < 0.05). Notably, PF-H treatment showed a superior modulatory effect on neurotransmitter homeostasis compared with the direct extract (DE-H), demonstrating a more pronounced elevation in 5-HT levels and the GABA/Glu ratio (*p* < 0.05) and a significant reduction in Glu levels (*p* < 0.05).

Histopathological evaluation of the hypothalamus by H&E staining (Figure 7) provided morphological observations complementary to the biochemical findings. Neurons in the NG exhibited regular morphology and an orderly arrangement. In contrast, the PCPA group showed apparent morphological abnormalities, including neuronal shrinkage, irregular cell size, and vacuole-like changes. Following treatment, these morphological abnormalities appeared less pronounced, and no visible vacuolar degeneration was observed. Although neuronal density in the extract-treated groups was slightly lower than that in the DZP group, the PF-H group appeared to show a relatively greater number of intact cells among the extract-treated groups. Overall, the H&E staining findings provide preliminary morphological observations that may be consistent with an attenuation of PCPA-associated hypothalamic histopathological changes following treatment, particularly with the high-dose fermented extract.

### 3.6. Modulation of Gut Microbiota by Co-Fermented Ganoderma lucidum Extract

#### 3.6.1. Effects on Microbial Diversity and Community Structure

To evaluate the effects of the extracts on the gut microbial community, 16S rRNA sequencing was performed on colon contents. The Venn diagram (Figure 8a) revealed 495 core operational taxonomic units (OTUs) shared among the NG, PCPA, DZP, DE-H, and PF-H groups. The PCPA model group exhibited fewer unique OTUs (1578) than the NG (1669). Notably, DZP, DE-H, and PF-H treatments resulted in 1669, 1743, and 1847 unique OTUs, respectively. The PF-H group had the highest number of unique OTUs, indicating greater microbial community richness following treatment with the co-fermented extract.

Alpha diversity was assessed using the Chao1 and Shannon indices (Figure 8b,c). Compared with the NG, PCPA administration significantly decreased both the Chao1 and Shannon indices (*p* < 0.05), indicating reduced microbial richness and diversity. Although DZP and DE-H treatments did not significantly affect the Chao1 index, the PF-H treatment showed an increasing trend. Furthermore, all three treatments significantly increased the Shannon index compared with the PCPA group (*p* < 0.05), with PF-H showing the greatest restorative effect on species evenness and diversity.

Beta diversity was assessed by principal coordinate analysis (PCoA; Figure 8d–g) to evaluate overall differences in gut microbial community structure. Bray–Curtis dissimilarities were evaluated using Adonis (PERMANOVA), which indicated that microbial community composition differed significantly between PCPA and NG (*p* = 0.002), DZP (*p* = 0.003), and PF (*p* = 0.035). In contrast, the PCPA–DE comparison did not reach statistical significance (*p* = 0.073). The PCoA plots demonstrated clear separation between the PCPA group and the NG and treatment groups. This separation indicates that PCPA administration significantly altered gut microbial community structure, whereas treatment with DZP, DE-H, and PF-H was associated with shifts in community structure toward that of the NG, although the patterns of these shifts differed among treatments.

#### 3.6.2. Taxonomic Composition and Identification of Microbial Biomarkers

To further characterize changes in microbial community structure, the relative abundances of the top 30 taxa at the genus level were analyzed (Figure 8i). The dominant genera across all groups primarily included *Lactobacillus, Muribaculum*, *Bacteroides*, unclassified *Muribaculaceae*, unclassified *Lachnospiraceae*, *Alloprevotella*, *Limosilactobacillus*, and *Eubacterium* (Group J). These taxa play important roles in maintaining intestinal microecological homeostasis.

Further intergroup analysis (Figure 8h) showed that PCPA-induced insomnia led to a significant decrease in the relative abundance of *Lactobacillus*, a beneficial genus widely associated with intestinal health. Treatment with DZP, DE-H, and PF-H significantly reversed this decrease, increasing *Lactobacillus* abundance (*p* < 0.05). Notably, PF-H showed a significantly greater increase in *Lactobacillus* abundance compared with the DE-H group (*p* < 0.05), suggesting that the observed restoration of this beneficial genus may be associated with co-fermentation.

To identify microbial biomarkers associated with these structural shifts, linear discriminant analysis effect size (LEfSe) analysis was performed using an LDA score threshold of >4.0 (Figure 8j). The NG was characterized by seven specific clades, including Campylobacterales and Helicobacteraceae. In contrast, the PCPA group was characterized by only one specific clade, *Ligilactobacillus*, indicating a marked alteration in its microbial profile. The DZP group was characterized by 11 specific clades, predominantly within the *Bifidobacterium* lineage. Notably, the direct extract (DE-H) yielded only one biomarker, *Limosilactobacillus*, whereas the fermented extract (PF-H) yielded seven taxonomic biomarkers, predominantly from the order *Lactobacillales*, mainly the genus *Lactobacillus*, including *Lactobacillus johnsonii*. The enrichment of these lactic acid bacteria, which have well-documented roles in gut health and the microbiota–gut–brain axis, may contribute to the enhanced sleep-promoting effects observed with the co-fermented extract [35].

## 4. Discussion

In this study, a novel enzyme-assisted bacteria–yeast co-fermentation system using *Lactobacillus acidophilus* and *Saccharomyces cerevisiae* was established to enhance the extraction of bioactive compounds from *Ganoderma lucidum*. The process was optimized by response surface methodology (RSM), and the resulting polysaccharide and triterpenoid yields (2.07% and 3.55%, respectively) exceeded those obtained by direct extraction (0.44% and 0.69%, respectively). Previous studies have employed ultrasound-assisted lactic acid bacterial fermentation [36] or prolonged fermentation with bifidobacteria [37]. Compared with these approaches, the present strategy achieved higher yields of both target components under the optimized conditions. The superior performance of this integrated strategy can be attributed to the synergistic effects between exogenous enzymatic pretreatment and microbial metabolism. Specifically, the initial treatment with cellulase, pectinase, and papain may facilitate the disruption of fungal tissue structures, which area primarily composed of chitin and glucans [18,19]. This “opening” step facilitated the mass transfer of intracellular components into the medium, thereby providing abundant substrates for the subsequent probiotic strains. We hypothesize that the exogenous enzymes primarily drove the depolymerization of macromolecules, whereas the co-fermentation process facilitated deeper biotransformation. For instance, the specific enrichment of triterpenoids such as ganoderic acid B, along with the evolution of sensory profiles (increased taste richness), is a characteristic outcome of complex microbial enzymatic activities rather than simple hydrolysis. Thus, the combination of enzymatic hydrolysis and bacterial-yeast fermentation offers a robust approach for overcoming the limitations of single-method processing.

Regarding the macromolecular components, despite the increase in the absolute polysaccharide yield, the relative mass fraction of polysaccharides in the fermented extract decreased. Although the Sevag method effectively removed most free proteins (resulting in a low protein content of 1.44%), the dilution effect likely occurred due to the co-precipitation of other non-carbohydrate materials during ethanol addition. Specifically, nucleic acids released from microbial lysis, unextractable bound complexes, and potentially the salts from the acetate buffer used during fermentation may have co-precipitated with the extracts. Nevertheless, this mixed macromolecular pool exhibited substantial changes in its physicochemical properties. The reduction in molecular weight and increased homogeneity suggest extensive depolymerization of the polysaccharides, consistent with previous findings [38]. Furthermore, monosaccharide composition and NMR analyses revealed a significant decrease in the relative abundance of Fuc and an increase in that of Man. Based on previous research on the selective utilization and transformation of polysaccharides by microorganisms [39], we hypothesize that the composite strains preferentially utilized Fuc-rich segments as carbon sources. Simultaneously, the significant increase in Man could result from a dual mechanism: the enzymatic release of Man from the *G. lucidum* polysaccharide backbone [40,41] and the accumulation of yeast mannans and microbial exopolysaccharides co-precipitated from the fermentation system [19,42]. Consequently, the fermented polysaccharides exhibited a more porous and less compact microstructure, which may have increased their bioaccessibility [43]. Given these complex interactions, the FT-IR, NMR, and SEM results presented in this study should be regarded as a preliminary characterization of the crude macromolecular pool rather than definitive structural evidence of a single purified polysaccharide. Ultimately, the observed microstructural and compositional changes reflect the synergistic outcome of fungal cell wall degradation, selective microbial utilization, and microbial macromolecule accumulation.

Beyond the macromolecular components, co-fermentation may alter the small-molecule metabolite profile of the extract. Untargeted metabolomics revealed increased abundances of several triterpenoids, particularly lucidenic acid B, which was not detected in the direct extract, accompanied by decreased abundances of ganoderic acid C2 and oleanolic acid. This suggests that microbial biotransformation may have contributed to the formation of lucidenic acid. Microbial proteolysis and biotransformation may have promoted the release or formation of umami-related amino acids, peptides, and nucleotides, contributing to the enhanced umami taste [44]. Meanwhile, the microbial degradation or transformation of bitterness-associated metabolites may have reduced their contribution to bitter perception, resulting in the observed decrease in bitterness and overall improvement in taste profile [45]. These chemical alterations may have contributed to the evolution of the sensory profile [46]. It is important to note that the sensory profiles obtained via the electronic tongue represent instrumental predictive variations based on chemical sensor responses. While these results suggest a potential reduction in bitterness and an enhancement in umami following fermentation, these instrumental findings do not directly equate to human consumer acceptance.

The aforementioned structural degradation of polysaccharides and targeted enrichment of bioactive triterpenes were associated with enhanced biological effects of the fermented extract. In vivo evaluation using a mouse model of PCPA-induced insomnia demonstrated that the fermented extract significantly attenuated body weight loss and prolonged pentobarbital-induced sleep duration, with greater effects than the direct extract. This enhanced efficacy may involve multiple mechanisms. Centrally, the fermented extract ameliorated neurotransmitter imbalances by increasing hypothalamic 5-HT and GABA levels and the GABA/Glu ratio, while reducing excitatory Glu levels [47]. Simultaneously, it modulated gut microbiota composition by increasing the abundance of *Lactobacillus* and reducing the abundance of *Prevotella*. Collectively, these findings suggest that the sleep-promoting effects of the fermented extract are associated with alterations in the gut microbiota and hypothalamic neurotransmitter levels, potentially involving the microbiota–gut–brain axis, providing a theoretical basis for further development of fermented *Ganoderma lucidum* as a functional food [48].

## 5. Conclusions

Co-fermentation of *Ganoderma lucidum* fruiting bodies with *Lactobacillus acidophilus* and *Saccharomyces cerevisiae* increased polysaccharide and triterpenoid yields and substantially altered the physicochemical properties, molecular-weight distribution, monosaccharide composition, and surface morphology of *Ganoderma lucidum* polysaccharides. Fermentation also altered the small-molecule profile and taste attributes of the ethanol extract. In mice with PCPA-induced insomnia, the high-dose fermented extract showed greater sleep-promoting activity than the non-fermented direct extract, as evidenced by reduced sleep latency, prolonged sleep duration, improved hypothalamic neurotransmitter homeostasis, alleviated neuronal damage, and changes in gut microbial diversity and composition. These findings suggest that enzyme-assisted bacteria–yeast co-fermentation may have potential for the development of *Ganoderma lucidum*-based sleep-promoting functional foods. Further work should focus on elucidating the fine structure of polysaccharide-rich fractions, validating and quantifying differential putative metabolites, and integrating gut microbiota and neurochemical analyses to clarify the material basis and explore a hypothesis regarding the potential involvement of the microbiota–gut–brain axis underlying the sleep-promoting activity of fermented *Ganoderma lucidum*.

## Figures and Tables

**Figure 1:**
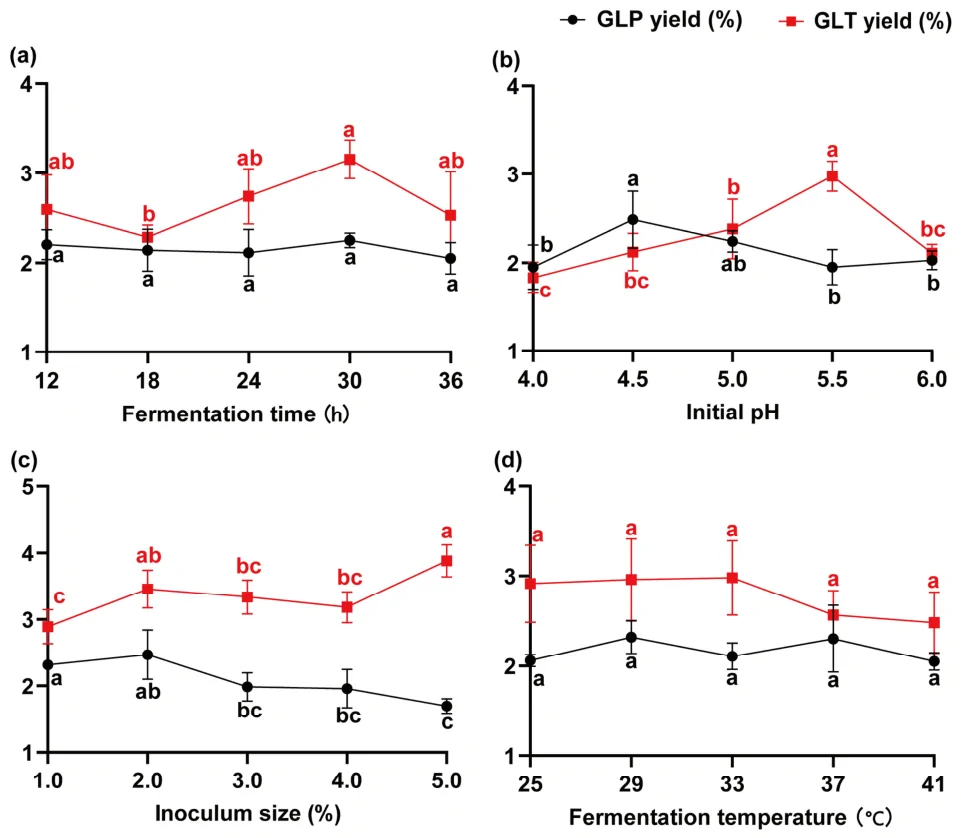
Effects of single-factor fermentation parameters on the yields of GLP and GLT: (**a**) fermentation time; (**b**) initial pH; (**c**) inoculum size; (**d**) fermentation temperature. Data are presented as mean ± standard deviation (SD) of triplicate determinations. Different lowercase letters indicate statistically significant differences among different conditions within the same indicator (*p <* 0.05).

**Figure 2:**
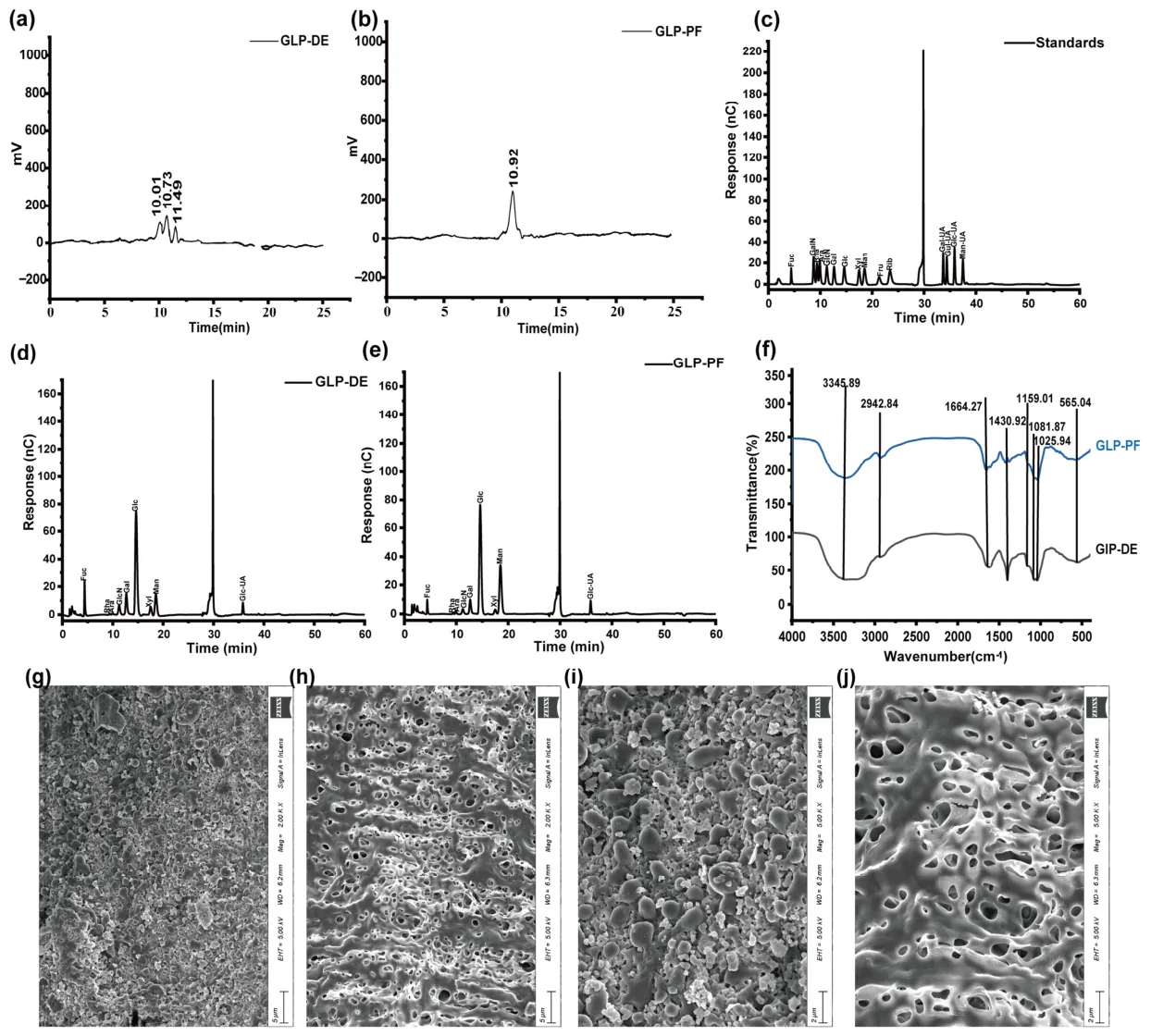
Preliminary structural characterization of the crude macromolecular fractions (GLP-DE and GLP-PF). (**a**,**b**) Apparent molecular weight distribution of GLP-DE and GLP-PF, respectively. (**c**–**e**) Ion chromatograms of monosaccharide standards, GLP-DE, and GLP-PF, respectively. (**f**) FT-IR spectra of GLP-DE and GLP-PF. (**g**–**j**) Scanning electron micrographs of the polysaccharides: (**g**) GLP-DE at 2000× magnification; (**h**) GLP-PF at 2000× magnification; (**i**) GLP-DE at 5000× magnification; (**j**) GLP-PF at 5000× magnification.

**Figure 3:**
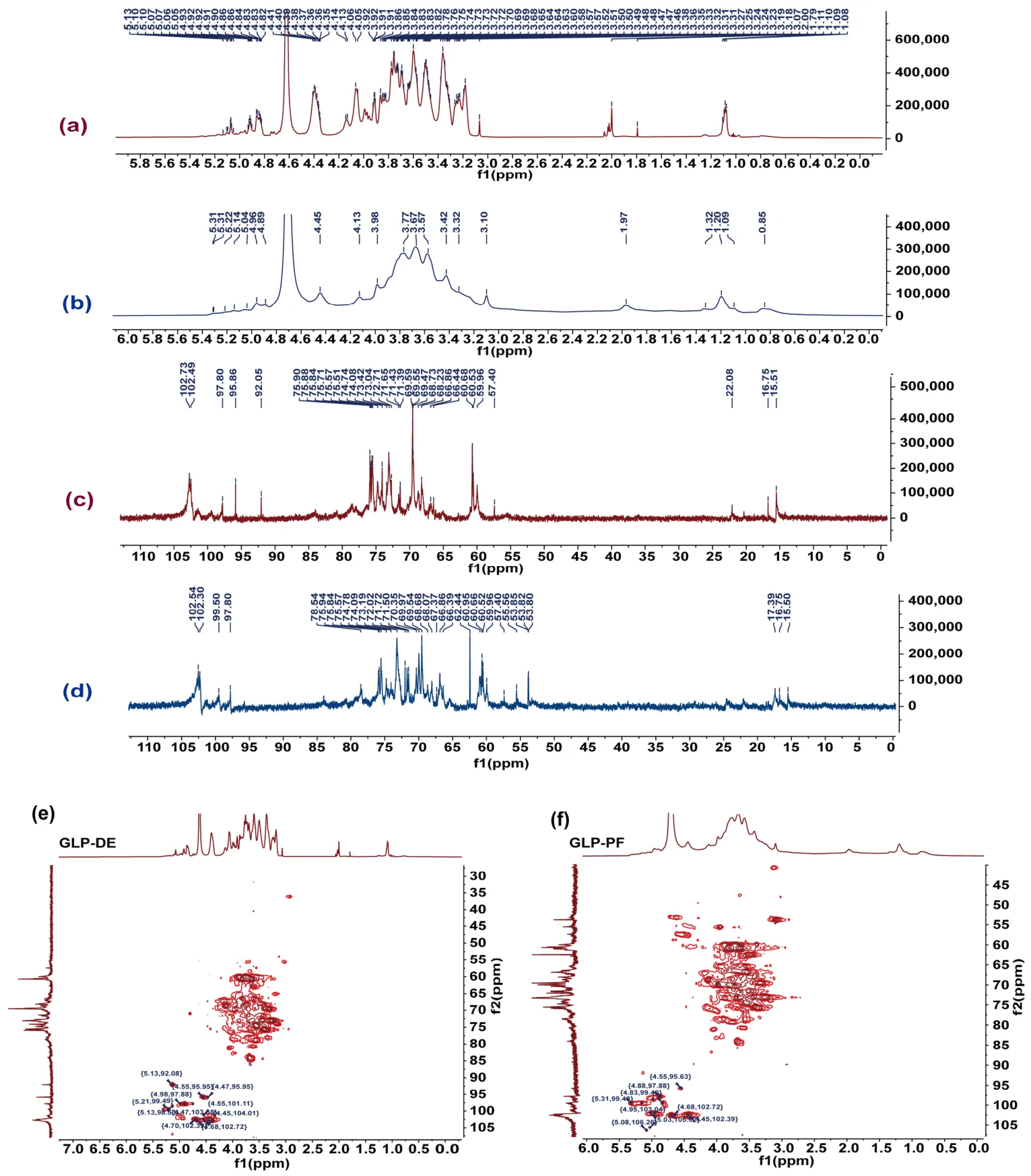
NMR spectra of crude polysaccharide fractions obtained via DE and PF. (**a**,**b**) 1H NMR spectra of GLP-DE and GLP-PF, respectively. (**c**,**d**) 13C NMR spectra of GLP-DE and GLP-PF, respectively. (**e**,**f**) 1H–13C HSQC two-dimensional NMR spectra of GLP-DE and GLP-PF, respectively.

**Figure 4:**
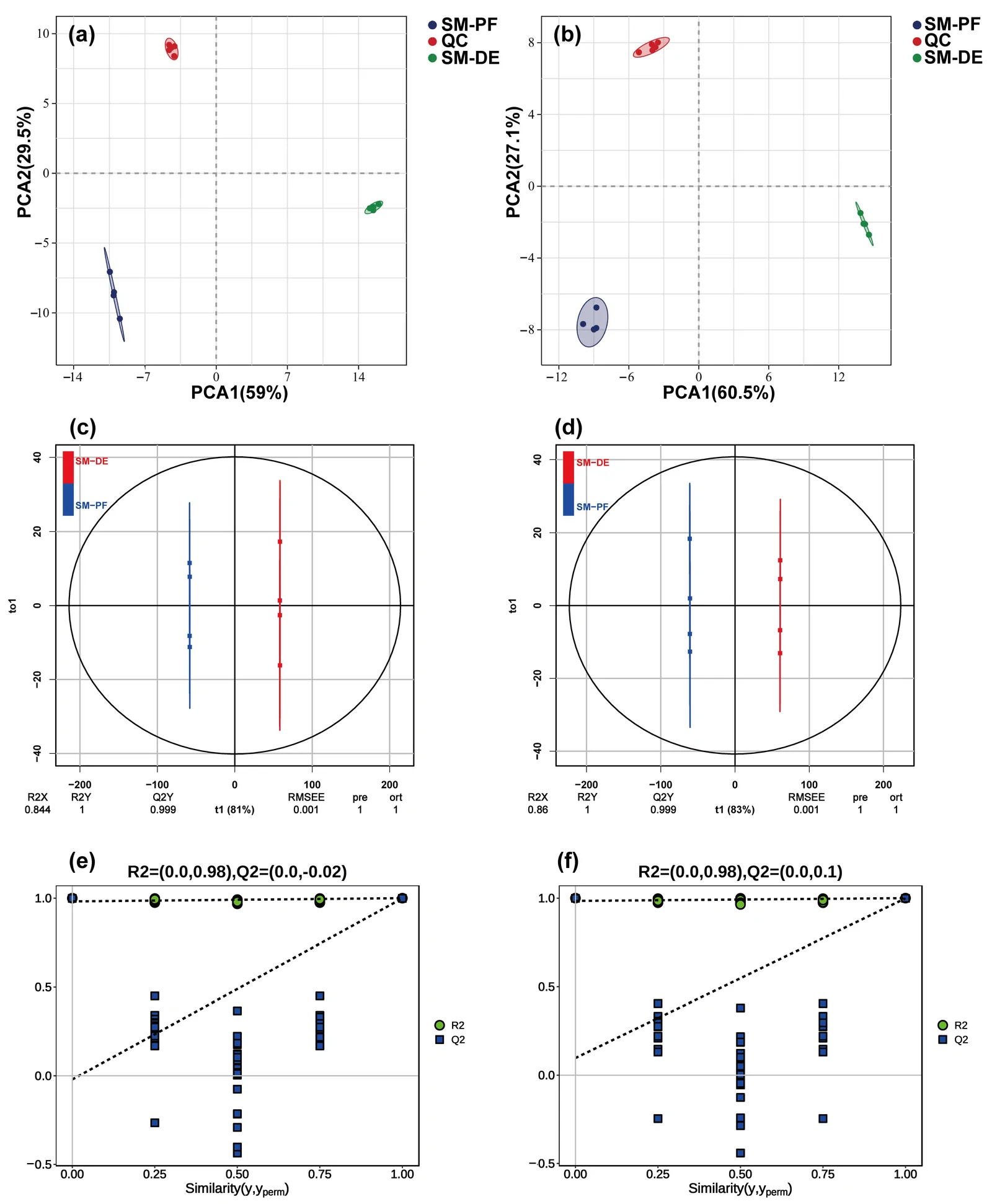
Multivariate statistical analysis of metabolite profiles obtained via direct extraction (SM-DE) and co-fermentation (SM-PF). (**a**,**b**) Principal component analysis (PCA) score plots in positive (ESI+) and negative (ESI−) ion modes, respectively. Quality control (QC) samples are included to evaluate system stability. (**c**,**d**) Orthogonal partial least squares-discriminant analysis (OPLS-DA) score plots comparing SM-DE and SM-PF in positive and negative ion modes, respectively. (**e**,**f**) Corresponding permutation test plots validating the OPLS-DA models in positive and negative ion modes, respectively.

**Figure 5:**
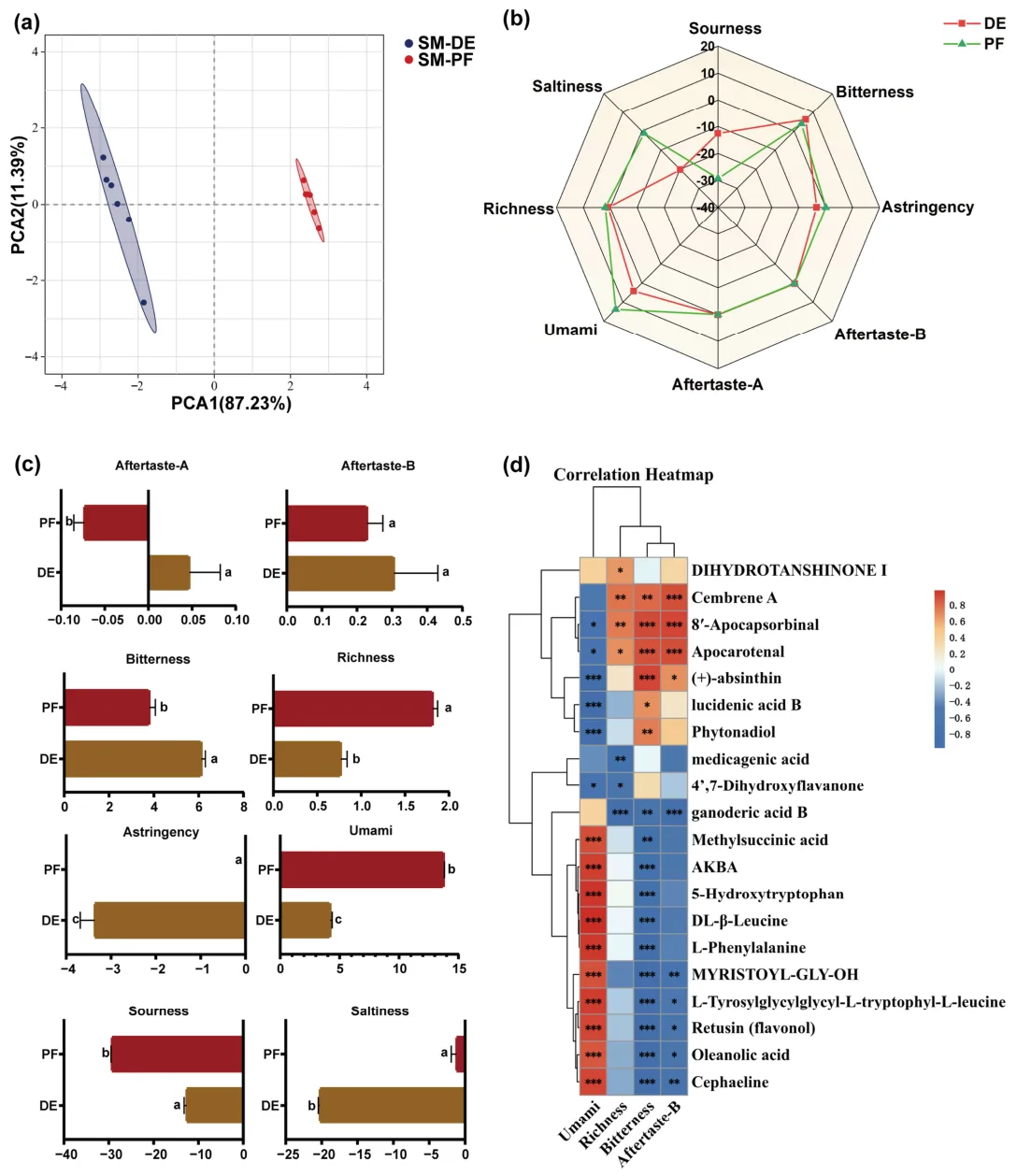
Flavor analysis of *Ganoderma lucidum* fruiting body extract and a diagram showing the correlation with the flavor profile of small molecules in the ethanol extract. Results of the Principal Component Analysis (PCA) of flavor components (**a**,**b**). Radar chart of flavor profiles (**c**). Different lowercase letters indicate statistically significant differences among groups (*p* < 0.05), whereas the same letters indicate no statistically significant differences. Analysis of significant differences in the five tastes (**d**) * *p* < 0.05, ** *p* < 0.01, *** *p* < 0.001.

**Figure 6:**
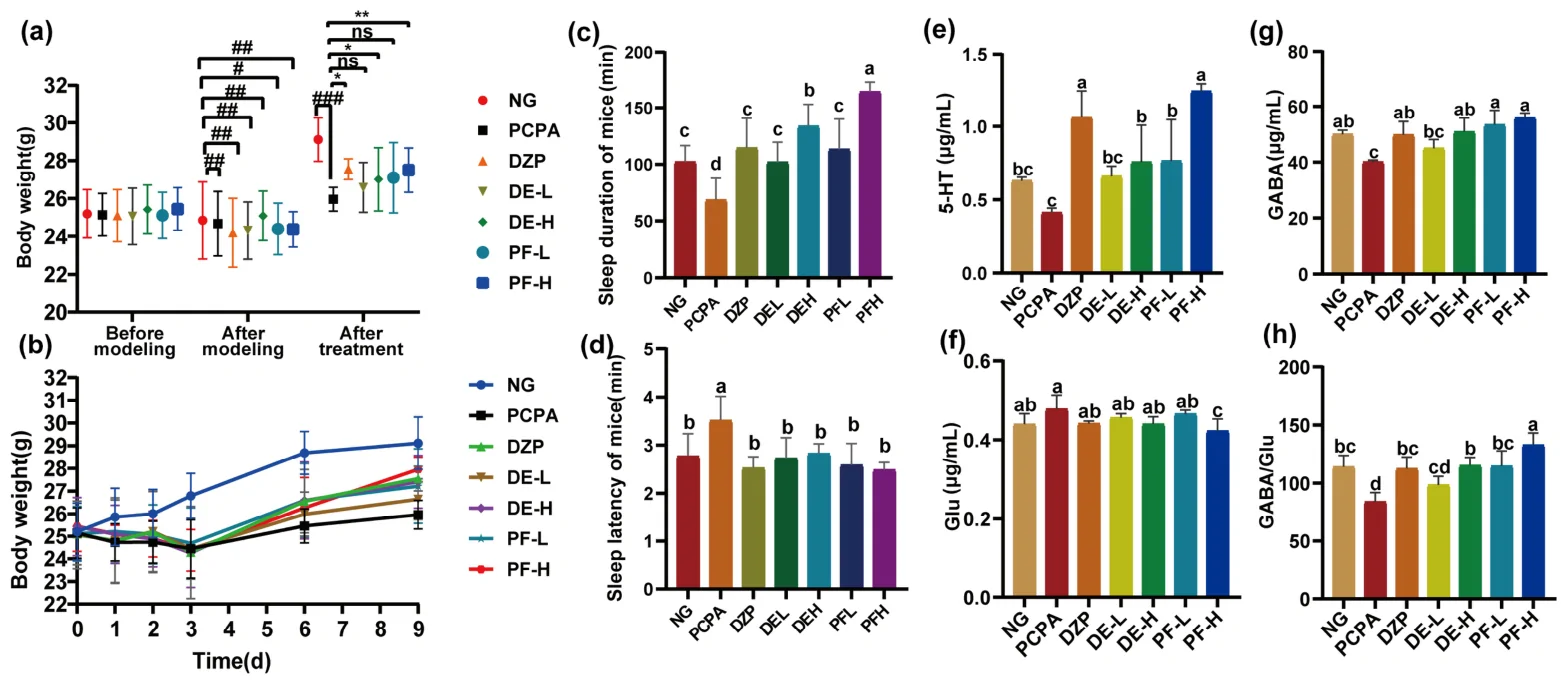
Body weight changes, sleep behavior and hypothalamic neurotransmitter levels of mice in each group ($\bar{x}\pm s$). (**a**) Body weight changes among groups before modeling (Day 0), after modeling (Day 3), and after administration (Day 9) (n = 8). (**b**) Trends of body weight changes in each group (n = 8). (**c**) Sleep duration of mice (n = 8). (**d**) Sleep latency of mice (n = 8). (**e**–**h**) Contents of 5-HT, GABA, and Glu and the GABA/Glu ratio in mouse hypothalamus (n = 3). # *p <* 0.05, ## *p <* 0.01 and ### *p <* 0.001 indicate statistically significant differences compared with the normal control group; * *p <* 0.05 and ** *p <* 0.01 indicate statistically significant differences compared with the model group. Different lowercase letters indicate statistically significant differences among groups (*p* < 0.05), whereas the same letters indicate no statistically significant differences.

**Figure 7:**
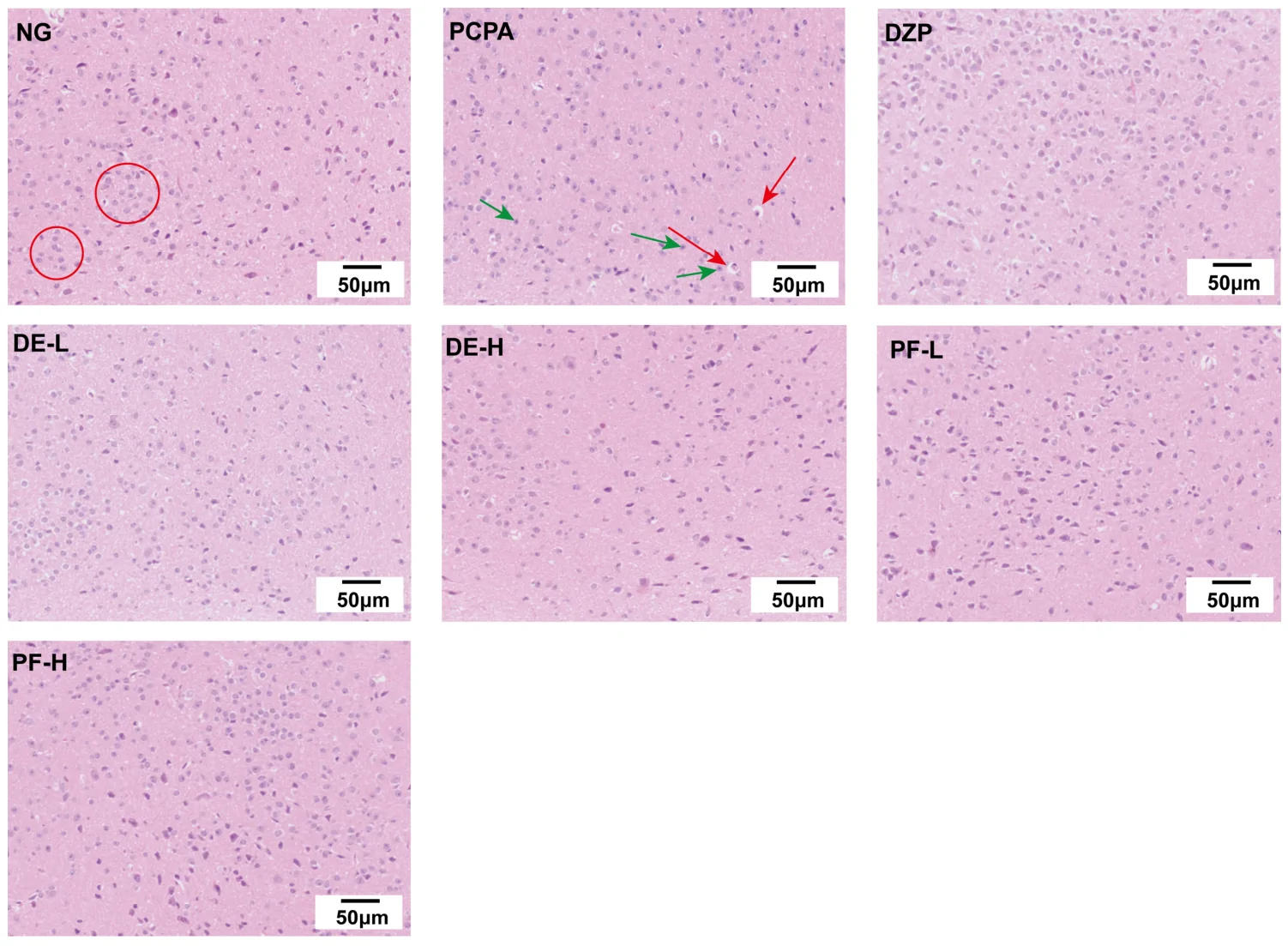
HE staining of mouse hypothalamic nerve cells (red circles indicate cells with regular morphology and distinct boundaries; green arrows indicate cytoplasmic loss and intensified nuclear staining; red arrows indicate cellular shrinkage and vacuolar degeneration).

**Figure 8:**
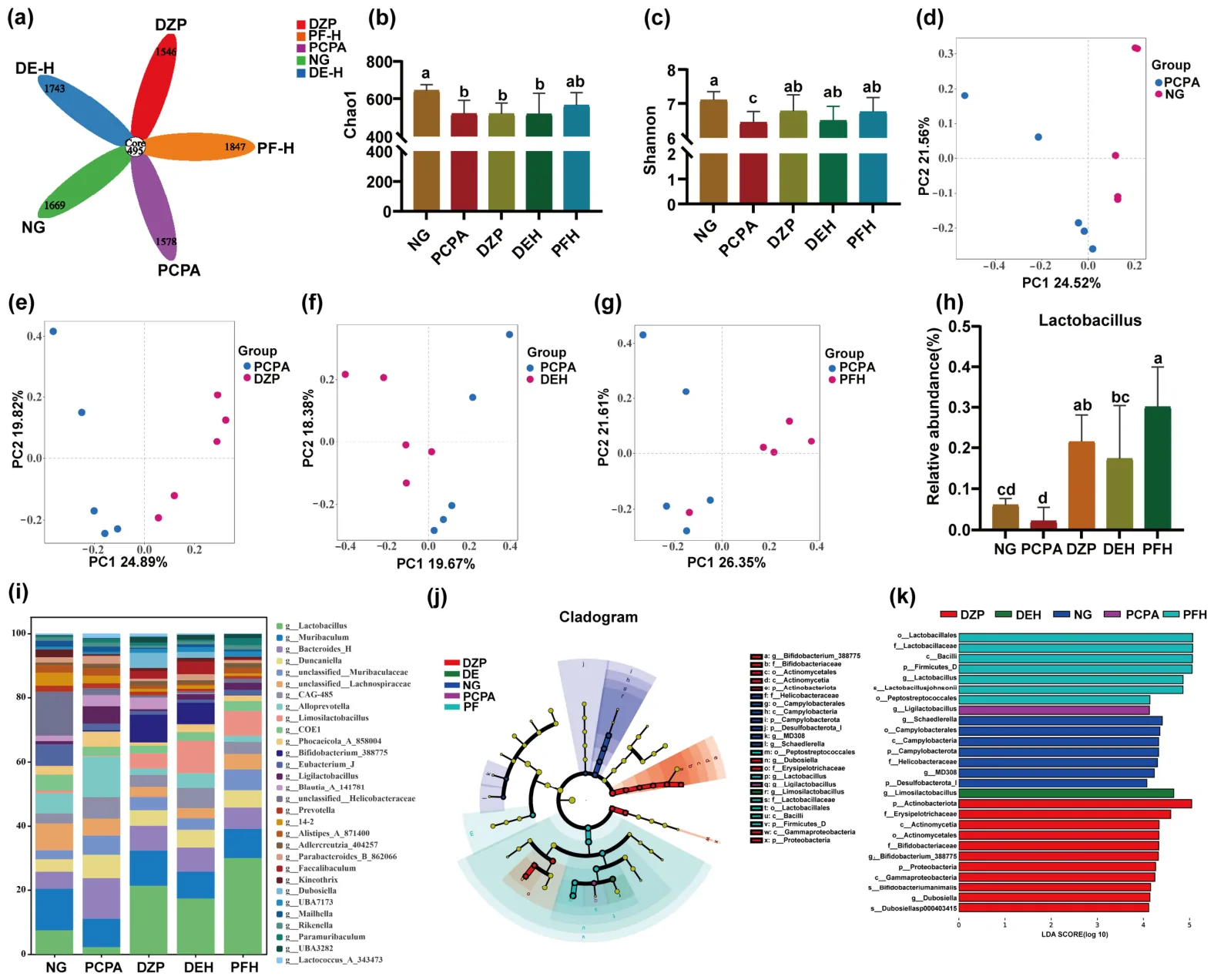
Analysis of gut microbiota composition and diversity in each group of mice (N = 6). (**a**) Representative petal plot; (**b**) Chao-1 index; (**c**) Shannon index; (**d**–**g**) β-diversity principal coordinate analysis plots; (**h**) relative abundance of *Lactobacillus*; (**i**) gut microbiota composition at the genus level; (**j**) phylogenetic trees for each group based on LEfSe analysis; (**k**) biomarker species score plots based on LEfSe analysis. Different lowercase letters indicate statistically significant differences among groups (*p* < 0.05), whereas the same letters indicate no statistically significant differences.

**Table 1:** Analysis of variance for the regression model of total polysaccharide yield.

Source	Sum of Squares	DF	Mean Square	*F*-Value	*p*-Value	Significance
Model	0.2204	9	0.0245	11.27	0.0021	Significant
A–Fermentation time	0.0055	1	0.0055	2.54	0.1552	Not significant
B–Initial fermentation pH	0.0190	1	0.0190	8.75	0.0211	Significant
C–Probiotic inoculum size	0.0578	1	0.0578	26.61	0.0013	Significant
AB	0.0072	1	0.0072	3.33	0.1110	Not significant
AC	0.0016	1	0.0016	0.7366	0.4192	Not significant
BC	0.0169	1	0.0169	7.78	0.0269	Significant
A^2^	0.0554	1	0.0554	25.52	0.0015	Significant
B^2^	0.0268	1	0.0268	12.33	0.0098	Significant
C^2^	0.0190	1	0.0190	8.77	0.0211	Significant
Residual	0.0152	7	0.0022			
Lack of fit	0.0097	3	0.0032	2.37	0.2120	Not significant
Pure error	0.0055	4	0.0014			
Total	0.2356	16				

**Table 2:** Analysis of variance for the regression model of total triterpene yield.

Source	Sum of Squares	DF	Mean Square	*F*-Value	*p*-Value	Significance
Model	3.22	9	0.3580	71.31	<0.0001	Significant
A–Fermentation time	0.0300	1	0.0300	5.98	0.0444	Significant
B–Initial fermentation pH	0.1275	1	0.1275	25.40	0.0015	Significant
C–Probiotic inoculum size	0.0032	1	0.0032	0.6374	0.4509	Not Significant
AB	0.0000	1	0.0000	0.0050	0.9457	Not significant
AC	0.0081	1	0.0081	1.61	0.2446	Not significant
BC	0.0064	1	0.0064	1.27	0.2961	Not Significant
A^2^	2.57	1	2.57	511.20	<0.0001	Significant
B^2^	0.2242	1	0.2242	44.65	0.0003	Significant
C^2^	0.0864	1	0.0864	17.21	0.0043	Significant
Residual	0.0351	7	0.0050			
Lack of fit	0.0278	3	0.0093	5.07	0.0755	Not significant
Pure error	0.0073	4	0.0018			
Total	3.26	16				

**Table 3:** Total sugar, uronic acid, and protein contents of GLP-DE and GLP-PF.

Group	Total Sugar Content (%)	Uronic Acid Content (%)	Protein Content (%)
GLP-DE	48.90 ± 3.96	5.63 ± 0.37	0.90 ± 0.08
GLP-PF	34.67 ± 0.73	8.93 ± 0.74	1.44 ± 0.38

## Data Availability

The original contributions presented in the study are included in the article/Supplementary Materials. Further inquiries can be directed to the corresponding authors.

## Supplementary Materials

The following supporting information can be downloaded at: https://www.mdpi.com/article/10.3390/foods15203716/s1, Figure S1: Total ion chromatograms (TICs) of SM-DE and SM-PF(A: ESI+, B: ESI−); Figure S2: Volcano plots of SM-DE/SM-PF(A: ESI+, B: ESI−); Table S1: Single-Factor Level Table; Table S2: Factors and levels based on Box–Behnken design; Table S3: Box–Behnken design with coded variables and observed responses; Table S4: Monosaccharide composition of *Ganoderma lucidum* fruiting body polysaccharides; Table S5: Information of differential metabolites in SM-PF/SM-DE.
