# Supplementary material for: Enzyme-Assisted Bacteria–Yeast Co-Fermentation of Ganoderma lucidum: Metabolomic Reprogramming, Sensory Evolution, and Sleep-Promoting Effects
Source: Foods. 2026 Oct 19;15(20):3716. doi: 10.3390/foods15203716 (PMC13704284; doi:10.3390/foods15203716)
Supplement: Supplementary file 1 [file foods-15-03716-s001.zip › foods-4612780-supplementary.pdf]

Supplementary Files

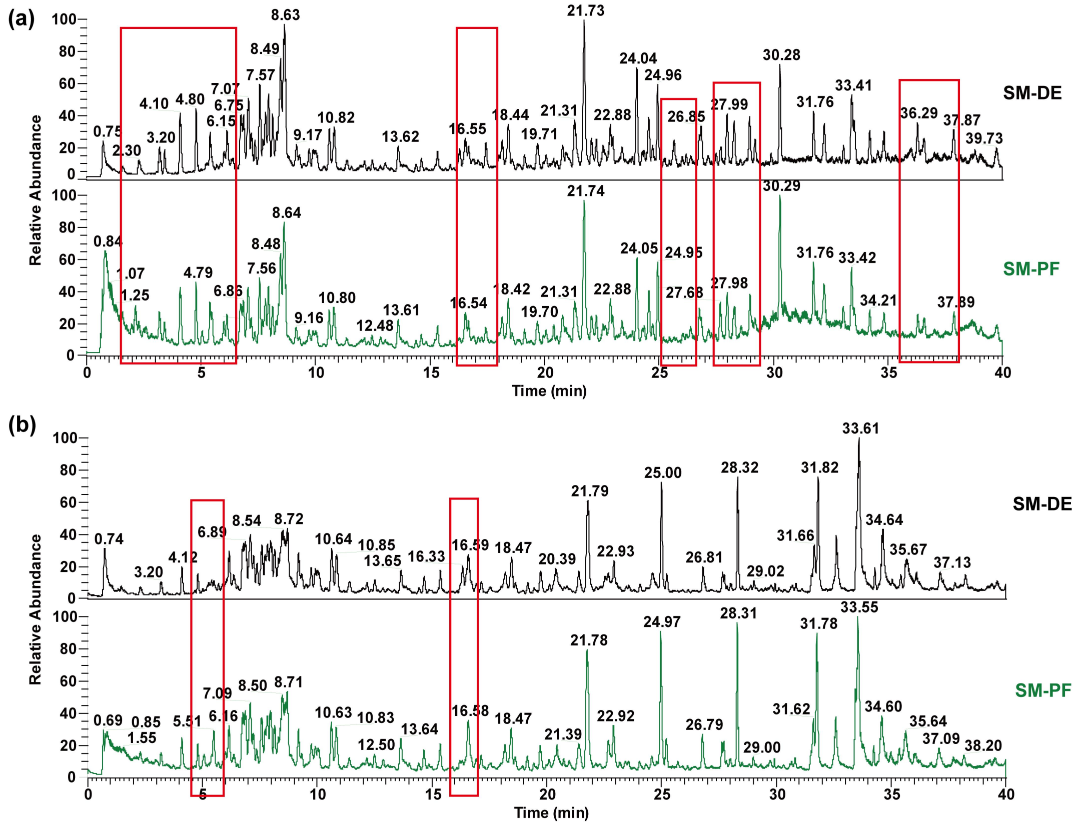

Figure S1. Total ion chromatograms (TICs) of SM-DE and SM-PF（A: ESI+, B: ESI−）

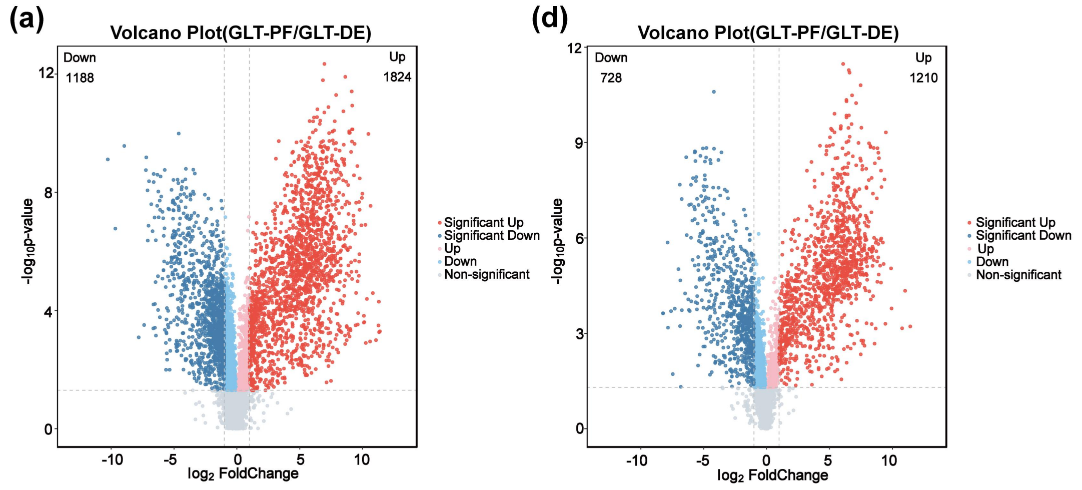

Figure S2. Volcano plots of SM-DE/SM-PF (A: ESI^{+}, B: ESI^{-})

Table S1. Single-Factor Level Table

Factors	Level Design
fermentation time（h）	12、18、24、30、36
fermentation temperature（℃）	25、29、33、37、41
initial pH	4.0、4.5、5.0、5.5、6.0
inoculum size（%，v/v）	1、2、3、4、5

Table S2. Factors and levels based on Box-Behnken design

Levels	Factors		
	A fermentation time（h）	B initial pH	C inoculum size(%)
-1	18	4.5	1
0	27	5.0	3
1	36	5.5	5

Table S3. Box-Behnken design with coded variables and observed responses

Run	A fermentation time（h）	B initial pH （pH）	C inoculum size（%）	GLP yield （%）	GLT yield （%）
1	27	5	3	2.11	3.68
2	18	5	5	1.87	2.61
3	27	5	3	2.1	3.63
4	36	5	5	1.8	2.82
5	18	5.5	3	2.02	2.65
6	27	5.5	5	1.91	3.40
7	27	5.5	1	2.01	3.53
8	36	5	1	1.95	2.76
9	27	5	3	2.08	3.71
10	27	5	3	2.02	3.60
11	27	4.5	1	2.07	3.08
12	36	4.5	3	1.82	2.64
13	27	4.5	5	1.71	3.11
14	18	5	1	1.94	2.73
15	27	5	3	2.05	3.65
16	36	5.5	3	1.86	2.77
17	18	4.5	3	1.81	2.51

Table S4 Monosaccharide composition of *Ganoderma lucidum* fruiting body polysaccharides

Sample	Fuc	Rha	Ara	GlcN	Gal	Glc	Xyl	Man	Glc-UA
GLP-DE（%）	13.13	0.40	0.31	5.63	9.86	51.72	3.76	12.28	2.90
GLP-PF（%）	5.01	0.33	0.91	2.74	6.30	52.10	1.98	27.45	3.18

Note: Fuc: fucose; Rha: rhamnose; Ara: arabinose; GlcN: glucosamine; Gal: galactose; Glc: glucose; Xyl: xylose; Man: mannose; Glc-UA: glucuronic acid.

Table S5 Information of differential metabolites in SM-PF/SM-DE

NO	HMDB ID	Name	Formula	m/z	RT [min]	Reference Ion	Log2 FC	P-value	Trend	Category
1	HMDB0245953	3-Acetyl-11-keto-β-boswellic acid	C32H48O5	513.35494	16.199	[M+H]+1	2.45	1.6188E-07	up	Triterpenoids
2	HMDB0037610	lucidenic acid B	C27H38O7	475.26878	6.005	[M+H]+1	1.1	1.1715E-05	up	Triterpenoids
3	HMDB0244078	DIHYDROTANSHINONE I	C18H14O3	277.0872	13.089	[M-H]-1	2.01	2.4203E-06	up	Diterpenoids
4	HMDB0245953	AKBA	C32H48O5	535.33934	23.409	[M+Na]+1	-0.09	0.37129278	down	Triterpenoids
5	HMDB0030579	Shikalkin	C16H16O5	289.10725	4.293	[M+H]+1	-0.67	0.00020845	down	Bicyclic Monoterpenoids
6	HMDB0035980	ganoderic acid E	C30H40O7	513.28466	7.489	[M+H]+1	-1.38	0.00088423	down	Triterpenoids
7	HMDB0004198	Phytonadiol	C31H48O2	453.37286	30.247	[M+H]+1	-1.95	7.0394E-07	down	Diterpenoids
8	HMDB0034517	18-β-Glycyrrhetinic acid	C30H46O4	469.33294	17.843	[M-H]-1	-1.88	0.00012029	down	Triterpenoids
9	HMDB0001645	L-Norleucine	C6H13NO2	132.10181	1.634	[M+H]+1	4.86	2.7615E-07	up	L-α-Amino Acids
10	HMDB0000472	5-Hydroxytryptophan	C11H12N2O3	221.08953	0.849	[M+H]+1	8.62	1.2425E-12	up	Amino Acids

NO	HMDB ID	Name	Formula	m/z	RT [min]	Reference Ion	Log2 FC	P-value	Trend	Category
11	HMDB0002339	5-Methoxytryptophan	C12H14N2O3	235.10759	1.195	[M+H]+1	4.35	0.00467581	up	L-Tryptophan Derivatives
12	HMDB0000159	L-Phenylalanine	C9H11NO2	166.08623	13.289	[M+H]+1	6.43	2.2781E-05	up	α-Amino Acids
13	HMDB0028823	L-γ-Glutamyl-L-leucine	C11H20N2O5	259.13001	1.336	[M-H]-1	7.12	3.4529E-08	up	Dipeptides
14	HMDB0041309	Monatin	C14H16N2O5	291.09886	1.082	[M-H]-1	2.32	0.00080566	up	α-Amino Acids
15	HMDB0241942	Stearoyl glutamic acid	C23H43NO5	412.30728	25.461	[M-H]-1	3.35	0.00014823	up	Amino Acids
16	HMDB0259343	Tyr-Ile-Gly-Ser-Arg	C26H42N8O8	595.31857	0.986	[M+H]+1	6.37	1.1309E-09	up	Oligopeptides
17	HMDB0259342	L-Tyrosylglycylglycyl-L-tryptophyl-L-leucine	C30H38N6O7	593.27341	11.977	[M-H]-1	4.45	0.0009154	up	Oligopeptides
18	HMDB0250791	DL-Phenylalanine	C9H11NO2	166.08621	6.527	[M+H]+1	4.97	6.5788E-06	up	Phenylalanine Derivatives
19	HMDB0028929	Gly-Leu	C8H16N2O3	171.11274	0.737	[M+H-H2O]+1	0.3	0.62223441	up	Dipeptides
20	HMDB0003640	DL-β-Leucine	C6H13NO2	130.08736	0.854	[M-H]-1	6.41	0.00055271	up	β-Amino Acids
21	HMDB0247190	Butyryl-L-homoserine lactone	C8H13NO3	172.09678	0.883	[M+H]+1	-4.52	7.6432E-08	down	N-Acyl-α-Amino Acids
22	HMDB0013272	N-lauroylglycine	C14H27NO3	258.20621	1.53	[M+H]+1	5.23	9.529E-08	up	Fatty Acids
23	HMDB0003581	dethiobiotin	C10H18N2O3	213.12454	2.009	[M-H]-1	6.15	8.5748E-06	up	Medium-Chain Fatty Acids

NO	HMDB ID	Name	Formula	m/z	RT [min]	Reference Ion	Log2 FC	P-value	Trend	Category
24	HMDB0000623	Dodecanedioic acid	C12H22O4	229.14466	3.942	[M-H]-1	1.68	1.4805E-05	up	Aliphatic Dicarboxylic Acids
25	HMDB0301804	12-Oxo phytodienoic acid	C18H28O3	293.21114	24.369	[M+H]+1	2.26	5.0343E-06	up	Medium-Chain Fatty Acids
26	HMDB0001844	Methylsuccinic acid	C5H8O4	131.035	0.789	[M-H]-1	5.29	0.00027056	up	Fatty Acids
27	HMDB0005066	C14-Carnitine	C21H41NO4	372.31077	24.55	[M+H]+1	4.38	6.98E-05	up	Acylcarnitines
28	HMDB0000222	Palmitoylcarnitine	C23H45NO4	400.34231	27.314	[M+H]+1	3.12	2.2149E-05	up	Acylcarnitines
29	HMDB0036634	Phloridzin	C21H24O10	437.14557	5.572	[M+H]+1	6.36	6.6759E-07	up	Flavonoid O-Glycosides
30	HMDB0000062	DL-Carnitine	C7H15NO3	162.11239	0.708	[M+H]+1	-7.03	1.3497E-07	down	Fatty Acids
31	HMDB0000821	Phenaceturic Acid	C10H11NO3	194.08127	1.361	[M+H]+1	-2.39	0.0011424	down	Fatty Acids
32	HMDB0010203	13(S)-HOTrE	C18H30O3	293.2126	19.5	[M-H]-1	-1.45	0.00264057	down	Linoleic Acids and Derivatives
33	HMDB0000893	Suberic acid	C8H14O4	173.082	2.693	[M-H]-1	-1.09	0.00453207	down	Fatty Acids
34	HMDB0030851	Aspalathin	C21H24O11	453.14058	5.593	[M+H]+1	6.18	1.187E-06	up	Flavonoids

NO	HMDB ID	Name	Formula	m/z	RT [min]	Reference Ion	Log2 FC	P-value	Trend	Category
35	HMDB0005760	4',7-Dihydroxyflavanone	C15H12O4	257.08081	7.71	[M+H]+1	-4.05	1.1202E-06	down	Flavonoids
36	HMDB0258335	5,2'-Dihydroxy-6,7,8,6'-tetramethoxyflavone	C19H18O8	375.10516	2.712	[M+H]+1	-4.74	1.7634E-06	down	Flavonoids
37	HMDB0037479	Xanthohumol	C21H22O5	355.15394	9.168	[M+H]+1	-2.61	0.0001871	down	Flavonoids
38	HMDB0038905	5,7,3',4'-Tetrahydroxy-6,8-diprenylisoflavone	C25H26O6	423.17804	12.76	[M+H]+1	-4.79	0.00236281	down	Isoflavonoids
39	HMDB0003306	Phloretin	C15H14O5	275.09143	3.053	[M+H]+1	-2.23	0.01258377	down	Dihydrochalcones
40	HMDB0005781	Glycitein	C16H12O5	285.07568	5.251	[M+H]+1	-4.82	2.27E-06	down	Isoflavones
41	HMDB0002670	(±)-Naringenin	C15H12O5	271.06146	2.762	[M-H]-1	-2.87	0.00644318	down	Flavonoids
42	HMDB0005810	Eriodictyol	C15H12O6	287.05646	3.492	[M-H]-1	-2.45	0.01376086	down	Flavanones
43	HMDB0132454	7,4′-Di-O-methylapigenin	C17H14O5	297.07725	3.704	[M-H]-1	-2.2	0.03692841	down	7-O-Methylated Flavonoids

NO	HMDB ID	Name	Formula	m/z	RT [min]	Reference Ion	Log2 FC	P-value	Trend	Category
44	HMDB0126497	Laricitrin	C16H12O8	331.04616	3.259	[M-H]-1	-4.08	3.8239E-06	down	Flavonols
45	HMDB0005801	Kaempferol	C15H10O6	285.0407	3.923	[M-H]-1	-1.26	0.00184365	down	Flavonols
46	HMDB0002780	Cianidanol	C15H14O6	291.08606	3.385	[M+H]+1	-1.92	0.00057837	down	Catechins
47	HMDB0038365	(+)-Gallocatechin	C15H14O7	305.06699	4.431	[M-H]-1	-5.12	7.0463E-07	down	Catechins
48	HMDB0037331	5,6-dimethoxy-2-(2-methoxyphenyl)-4H-chromen-4-one	C18H16O5	313.10452	3.475	[M+H]+1	-5.6	0.00020048	down	6-O-Methylated Flavonoids
49	HMDB0302531	Byakangelicol	C17H16O6	315.08757	5.079	[M-H]-1	3.09	2.1981E-05	up	5-Methoxypsoralen
50	HMDB0302525	8,8-dimethyl-2H,8H-pyrano[3,2-g]chromen-2-one	C14H12O3	229.08353	2.678	[M+H]+1	6.15	9.8356E-07	up	Linear Furanocoumarins
51	HMDB0030651	Byakangelicin	C17H18O7	335.11257	1.916	[M+H]+1	-4.57	1.191E-07	down	5-Methoxypsoralen
52	HMDB0013695	Urolithin A	C13H8O4	229.04954	7.319	[M+H]+1	-2.16	2.7757E-05	down	Coumarins and Derivatives
53	HMDB0030853	iso-Meranzin	C15H16O4	261.11215	16.321	[M+H]+1	-2.34	5.3707E-05	down	Coumarins and Derivatives

NO	HMDB ID	Name	Formula	m/z	RT [min]	Reference Ion	Log2 FC	P-value	Trend	Category
54	HMDB0034376	Imperatorin	C16H14O4	271.09663	11.085	[M+H]+1	-4.73	1.8043E-06	down	Psoralens
55	HMDB0250923	Decursin	C19H20O5	329.13843	10.094	[M+H]+1	-1.97	0.00042111	down	Linear Furanocoumarins
56	HMDB0256020	(+)-Oxypeucedanin hydrate	C16H16O6	303.0875	4.809	[M-H]-1	-2.26	0.00202344	down	Psoralens
57	HMDB0302150	Osthol	C15H16O3	243.10284	3.436	[M-H]-1	-1.07	8.2212E-05	down	Coumarins
58	HMDB0250486	Coumafuryl	C17H14O5	297.07713	3.514	[M-H]-1	-2.51	1.334E-05	down	4-Hydroxycoumarin
59	HMDB0030311	Harmine	C13H12N2O	213.10222	1.556	[M+H]+1	2.85	1.3634E-05	up	Alkaloids
60	HMDB0030310	Harmaline	C13H14N2O	215.11774	5.426	[M+H]+1	4.4	7.1607E-07	up	Alkaloids
61	HMDB0249806	Cephaeline	C28H38N2O4	467.29246	28.257	[M+H]+1	1.51	0.00558719	up	Alkaloids
62	HMDB0033731	leucoline	C9H7N	130.06495	3.77	[M+H]+1	5.48	4.0047E-06	up	Alkaloids
63	HMDB0015245	papaverine	C20H21NO4	340.15414	6.327	[M+H]+1	-4.49	1.8999E-06	down	Alkaloids

NO	HMDB ID	Name	Formula	m/z	RT [min]	Reference Ion	Log2 FC	P-value	Trend	Category
64	HMDB0248144	Alizarin	C14H8O4	241.04955	4.658	[M+H]+1	2.2	0.02696172	up	Hydroxyanthraquinones
65	HMDB0035214	Emodin	C15H10O5	271.06006	7.179	[M+H]+1	-4.29	2.4341E-07	down	Hydroxyanthraquinones
66	HMDB0255722	Norathyriol	C13H8O6	259.02502	2.238	[M-H]-1	-3.32	0.00513266	down	Xanthones
67	HMDB0033332	Garcinone D	C24H28O7	427.1744	9.059	[M-H]-1	-4.74	0.00017124	down	Xanthones
68	HMDB0250583	Cucurbitacin S	C30H42O6	499.30562	10.591	[M+H]+1	-1.28	0.00073462	down	Steroids
69	HMDB0259897	Withaferine	C28H38O6	469.26002	17.549	[M-H]-1	-5.3	0.0004305	down	Steroids
70	HMDB0033846	Erisimin	C29H42O9	533.27559	9.022	[M-H]-1	-2.78	0.00024218	down	Steroids
71	HMDB0030174	Tetrandrine	C38H42N2O6	623.31297	1.289	[M+H]+1	7.28	6.2647E-09	up	Lignans
72	HMDB0341205	(+)-Pinoresinol	C20H22O6	359.14896	6.517	[M+H]+1	4.33	4.2596E-07	up	Furofuran Lignans
73	HMDB0253312	hypophyllanthin	C24H30O7	429.19007	9.465	[M-H]-1	-5.44	3.9804E-06	down	Lignans

NO	HMDB ID	Name	Formula	m/z	RT [min]	Reference Ion	Log2 FC	P-value	Trend	Category
74	HMDB0256643	Plicatic acid	C20H22O10	421.11442	2.718	[M-H]-1	-1.71	9.0966E-06	down	Lignans
75	HMDB0005807	Gallic acid	C7H6O5	169.01428	1.386	[M-H]-1	-0.7	0.06752984	down	Gallic Acid
76	HMDB0254590	Methyl gallate	C8H8O5	183.02995	1.253	[M-H]-1	-2.07	1.8695E-06	down	Gallate Esters
77	HMDB0002899	Ellagic acid	C14H6O8	300.9992	6.983	[M-H]-1	-5.12	2.0431E-05	down	Tannins
78	HMDB0037642	Butyl vanillyl ether	C12H18O3	209.11844	17.62	[M-H]-1	-4.74	9.2736E-06	down	Methoxyphenols
79	HMDB0242211	Haematoxylin	C16H14O6	301.07208	2.926	[M-H]-1	-2.76	0.01647816	down	Benzopyrans
80	HMDB0030791	(+)-dihydromethysticin	C15H16O5	257.08215	1.925	[M-H-H2O]-1	-4.3	1.0149E-07	down	Kavalactones
81	HMDB0254337	Marinobufagenin	C24H32O5	401.23208	6.728	[M+H]+1	1.54	0.0108087	up	Butenolides
82	HMDB0038419	Glucotropeolin	C14H19NO9S2	408.04294	0.858	[M-H]-1	5.44	0.01573405	up	Alkyl Thioglucosides
83	HMDB0250754	D-Glucosamine 6-phosphate	C6H14NO8P	260.05298	0.716	[M+H]+1	-3.16	5.7302E-05	down	Monosaccharide Phosphates

NO	HMDB ID	Name	Formula	m/z	RT [min]	Reference Ion	Log2 FC	P-value	Trend	Category
84	HMDB0000244	Riboflavin	C17H20N4O6	375.13104	1.463	[M-H]-1	-1.8	1.283E-07	down	Flavins
85	HMDB0254201	LUMIFLAVIN	C13H12N4O2	255.08877	1.456	[M-H]-1	-1.51	0.00328732	down	Flavins
86	HMDB0033616	10-GINGEROL	C21H34O4	349.23871	19.942	[M-H]-1	-5.85	1.311E-08	down	Gingerols
87	HMDB0302135	Hexahydrocurcumin	C21H26O6	375.17986	6.817	[M+H]+1	-1.11	0.0038986	down	Curcuminoids
88	HMDB0005789	Tetrahydrocurcumin	C21H24O6	371.1501	9.193	[M-H]-1	-2.37	0.00012985	down	Curcuminoids
89	HMDB0258333	4,7,8-trimethoxyfuro[2,3-b]quinoline	C14H13NO4	260.08927	1.437	[M+H]+1	6.92	4.3779E-07	up	Furoquinolines
90	HMDB0001896	5-Methoxytryptophol	C11H13NO2	192.10186	2.773	[M+H]+1	4.39	0.00106989	up	5-Methoxytryptophan Phenols
91	HMDB0015224	Ouabain	C29H44O12	585.29254	7.885	[M+H]+1	-1.43	0.01448838	down	Glycosides
92	HMDB0250592	Curculigoside A	C22H26O11	467.15624	6.515	[M+H]+1	5.15	1.8163E-05	up	Phenolic Aldehydes
93	HMDB0303171	Isorhapontigenin	C15H14O4	259.0965	8.537	[M+H]+1	-3.7	1.1692E-08	down	Stilbenes

NO	HMDB ID	Name	Formula	m/z	RT [min]	Reference Ion	Log2 FC	P-value	Trend	Category
94	HMDB0002302	IpA	C11H11NO2	190.08626	1.14	[M+H]+1	-2.66	5.7521E-05	down	Indoles and Derivatives
95	HMDB0000881	Xanthurenic acid	C10H7NO4	204.03035	2.588	[M-H]-1	-4.06	0.00557945	down	Quinolines
96	HMDB0341316	Fraxinellone	C14H16O3	231.10277	4.861	[M-H]-1	-1.78	0.00549763	down	Isobenzofurans
97	HMDB0250954	Dehydroevodiamine	C19H15N3O	300.11442	9.886	[M-H]-1	5.71	1.8064E-06	up	Quinazolines
98	HMDB0004062	2,5-Dihydroxybenzaldehyde	C7H6O3	137.02443	0.799	[M-H]-1	-3.79	2.2939E-06	down	Hydroxybenzaldehydes
